# Detecting Convergence of Amino Acid Physicochemical Properties Underlying the Organismal Adaptive Convergent Evolution

**DOI:** 10.1111/1755-0998.70052

**Published:** 2025-09-26

**Authors:** Shanshan Chen, Zhengting Zou

**Affiliations:** ^1^ State Key Laboratory of Animal Biodiversity Conservation and Integrated Pest Management Institute of Zoology, Chinese Academy of Sciences Beijing China; ^2^ University of Chinese Academy of Sciences Beijing China

**Keywords:** adaptive convergence, amino acid physicochemical property, convergent evolution, radical substitution, sequence convergence

## Abstract

Many studies have proposed various comparative genomic methods to probe the molecular basis for adaptive functional convergence between species, conventionally by detecting the convergence of amino acid states between orthologous protein sequences of these species or lineages. However, different amino acids with similar physicochemical properties at a site may contribute to the functional similarity of the protein. Hence, could the convergence of amino acid physicochemical properties, in addition to state convergence, also contribute to adaptive convergence of organismal functions? Here we grouped amino acids into physicochemically similar classes, and developed computational pipelines to detect the Convergence of Amino Acid Properties (CAAP, https://github.com/shanschen33/CAAP) by modifying previous state convergence detection methods. Investigating three organismal convergence cases including echolocating mammals, marine mammals and woody mangroves, we found genes with CAAP that likely contribute to the respective functional adaptation, supported by orthogonal evidence such as functional enrichment and positive selection analyses. Our findings in multiple cases corroborate the hypothesis that CAAP may underlie adaptive convergent evolution of organismal functions, emphasising the importance of considering sequence features more complex than amino acid states when studying adaptive sequence convergence.

## 1 Introduction

In evolutionary biology, convergent evolution, or convergence, is defined as the independent emergence of similar or identical traits in two or more lineages of species. Since the repeated emergence of the same complex trait is unlikely random, organism‐level functional convergence is often regarded as evidence of adaptive evolution. For example, the development of echolocation ability in some bat species and toothed whales is likely adaptive to their dim‐light living environment (Madsen and Surlykke 2013; Stern 2013). In recent years, studies have extensively explored convergence at the molecular sequence level, aiming to identify key sites or genes that underlie various cases of adaptive functional convergence in different organisms (Parker et al. 2013; Liu et al. 2014; Foote et al. 2015; Natarajan et al. 2015; Chikina et al. 2016; Xu et al. 2017; He et al. 2020; Fukushima and Pollock 2023). For example, a convergent asparagine‐to‐threonine substitution that happened at position 7 of the protein *Prestin* in both echolocating bats and toothed whales has been related to increased high‐frequency sound perception ability (Li et al. 2010; Liu et al. 2014). However, sequence‐level convergence may well result from non‐adaptive, neutral evolutionary processes (Zhang and Kumar 1997; Zhang 2006; Goldstein et al. 2015; Storz 2016). Hence, it has been proposed that an excessive amount of sequence convergence beyond the neutral evolution expectation may indicate the molecular basis for adaptive functional convergence (Zhang 2006; Zou and Zhang 2015b). Many studies have developed varying strategies to detect such non‐random signals in comparative sequence data (Zhang and Kumar 1997; Castoe et al. 2009; Li et al. 2010; Foote et al. 2015; Thomas and Hahn 2015; Zou and Zhang 2015a; Xu et al. 2017; He et al. 2020; Barteri et al. 2023; Fukushima and Pollock 2023; Allard et al. 2025). Notably, all of these methods focus on the events of state convergence, that is, the independent emergence of the same amino acid state in two or more species lineages of interest. In addition, several other studies detect adaptive sequence convergence beyond amino acid states, for example, by tracing the convergent changes of amino acid frequency profiles (Rey et al. 2018) or those of sequence evolution rates (Prudent et al. 2016; Hu et al. 2019; Kowalczyk et al. 2019; Treaster et al. 2021).

Nonetheless, the 20 amino acids composing natural proteins can be grouped into physicochemically similar classes. For example, arginine (R), histidine (H) and lysine (K) are all positively charged (Zhang 2000). Studies have shown that the physicochemical changes introduced by different amino acid substitutions dominate protein evolution patterns (Tang et al. 2004; Chen, He, et al. 2019; Chen, Lan, et al. 2019). Generally, radical amino acid changes between different physicochemical classes introduce stronger fitness effects and are more likely to underlie adaptive evolution. In contrast, amino acids within each class are considered interchangeable to some extent, so within‐class changes cause smaller functional disturbance to the protein (Zhang 2000; Hanada et al. 2007; Lin 2008; Lyons and Lauring 2017). However, the conventional methods detecting amino acid state convergence underrate this factor at least in two situations: (1) they do not explicitly consider the different potential of radical and conservative changes in causing adaptive functional changes; and (2) they do not consider the possible cases where independent amino acid changes to two different but physicochemically similar amino acids can be functionally convergent. In situation (1), conservative changes may happen more frequently but are more likely to be neutral noise, as reasoned above. In situation (2), putatively adaptive signals are neglected. Indeed, it has been proposed that noise should be reduced in the detection of convergence to reveal valid signals (Wu et al. 2020). A potential solution to these problems is to focus on the site‐wise physicochemical property changes during protein sequence evolution. Hence, could we detect convergence of amino acid physicochemical properties, in addition to amino acid state convergence, to be the molecular basis of adaptive functional convergence at the organismal level?

Here, we defined the convergence of amino acid properties (CAAP) and revisited three existing cases of convergence (echolocating mammals, marine mammals and woody mangroves) in different focal species with different methodologies for detecting state convergence. We detected genes with CAAP in each case by modifying the corresponding original methods for detecting state convergence. To explore the function of these genes, we performed functional enrichment analyses including GO enrichment and KEGG pathway enrichment. For echolocating mammals, we also conducted positive selection tests for all the detected genes with CAAP. Additionally, an extensive literature review of the CAAP genes provided further evidence supporting our results. Consequently, we uncovered a considerable number of candidate genes with putative adaptive property convergence in each case. These genes were enriched in biological functions and pathways relevant to ecological adaptation. Besides, some genes were found to be under positive selection and have been associated with adaptive convergent evolution by previous computational or experimental studies.

## 2 Materials and Methods

### 2.1 Collect Protein Sequence Data

**FIGURE 1:**
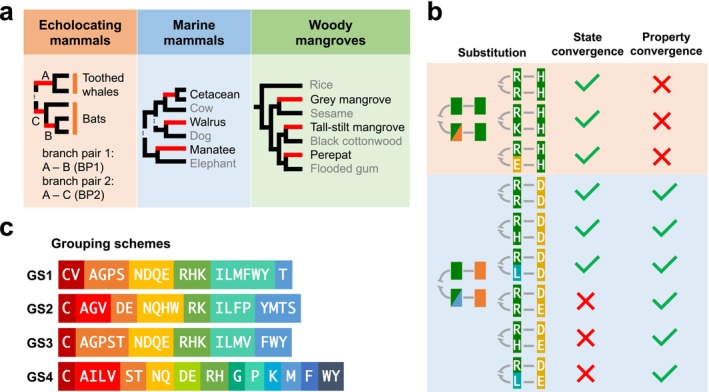
Revisiting organismal function convergence cases by focusing on the convergence of amino acid physicochemical properties, rather than amino acid state. (a) Simplified phylogenies of three cases with organismal function convergence. Leaf names in black indicate taxa with the focal functions such as echolocation, whereas names in grey are background taxa. Focal branches in the analyses are coloured in red. (b) Schematic cases of the conventional amino acid state convergence and the property convergence (CAAP) proposed in this work. Convergence cases can be divided into exclusive state convergence, ‘property and state’ convergence, and exclusive property convergence. (c) Four grouping schemes (GSs) used in the analyses. Distinct colour backgrounds indicate amino acid classes with different physicochemical properties.

**FIGURE 2:**
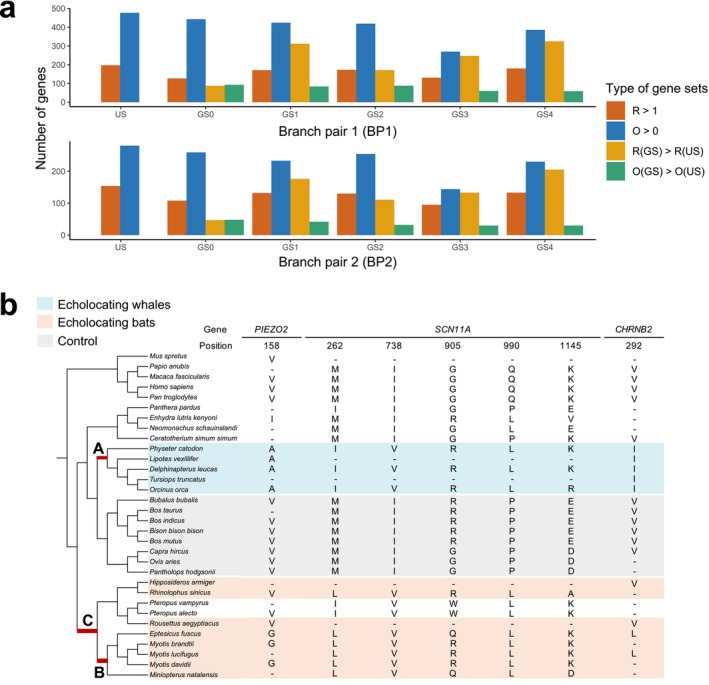
Genes exhibiting CAAP are functionally related to organismal convergence in echolocating mammals. (a) The numbers of genes with *R* > 1, O > 0, R(GS) > R(US) and O(GS) > O(US) under each grouping scheme (US or GS0‐4) between different branch pairs (BP). (b) Tree topology and alignment of all sites with state or property convergence in three example genes. The taxon names and corresponding amino acid states of toothed whales and echolocating bats are shaded in blue and orange, respectively. Focal branches A, B, C are highlighted in red. The taxon names and corresponding amino acid states of eight bovid species used as negative control are shaded in grey. Two non‐echolocation bats and another 10 species that appeared most frequently in all gene alignments were also included as background taxa. For clarity, we used only a subset of all 116 mammal species in the figure, whereas the complete species tree included in the analysis is shown in Figure S1. Numbers above each site indicate position indices in the filtered protein sequence alignments. The character ‘‐’ indicates that the sequence of the corresponding species did not participate in the analysis after data filtering.

One‐to‐one orthologous protein sequences of all available species (up to 116) for 14,509 genes were downloaded from OrthoMaM v10c (Scornavacca et al. 2019; https://www.orthomam.univ‐montp2.fr/orthomam_v10b/cds/archives/omm_AA_fasta.v10c_116tax_CDS_final.tar.gz) for the analysis of echolocating mammals, including the echolocating Yinpterochiroptera bat species ( *Hipposideros armiger* , *Rhinolophus sinicus* , *Rousettus aegyptiacus* ), echolocating Yangochiroptera bat species ( *Eptesicus fuscus* , *Miniopterus natalensis* , *Myotis brandtii* , *Myotis davidii* , *Myotis lucifugus* ) and the toothed whales ( *Physeter catodon* , *Lipotes vexillifer* , *Orcinus orca* , *Delphinapterus leucas* , *Tursiops truncatus* ); and for the analysis of marine mammals ( *Odobenus rosmarus* , *Trichechus manatus latirostris* , *Tursiops truncatus* , *Orcinus orca* ). To detect convergence in echolocating mammals, we adopted the species tree topology in OrthoMaM (Figure S1) and investigated two pairs of branches (Figure 1a) according to different evolutionary hypotheses (Sulser et al. 2022): (1) the basal branch of the toothed whales and the basal branch of Yangochiroptera (BP1, branch A and B in Figure S1 and Figure 1a); (2) the basal branch of the toothed whales and the basal branch of all bats (BP2, branch A and C in Figure S1 and Figure 1a). We selected branch B as a focal branch because it leads to the yangochiropterans which echolocate using broadband frequency‐modulated (FM) calls in short, widely spaced pulses, known as low duty cycle (LDC) echolocation (Sulser et al. 2022). Oppositely, the yinpterochiropterans make narrowband or constant frequency sounds in long, shortly spaced pulses, referred to as high duty cycle (HDC) echolocation. The previous study has experimentally proven that LDC echolocation is a more ancestral trait compared to HDC (Sulser et al. 2022). Meanwhile, short, broadband clicks are also prevalent and considered ancestral in toothed whales (Brinkløv et al. 2022). In addition, several studies have investigated the convergent evolution of echolocation in bats or between bats and other organisms (e.g., birds) by selecting this branch as one focal branch (Teeling et al. 2016; Nojiri et al. 2021; Sadanandan et al. 2023). We required that a protein alignment contain at least one Yinpterochiroptera species, more than one Yangochiroptera species and more than one toothed whale species to be included in downstream analyses. This step is to ensure that the resulting protein alignments can form the two branch pairs we mentioned above. Regarding marine mammals, to maintain consistency with the original study (Foote et al. 2015), we retained protein alignments with at least the four focal species with their nearest non‐marine species and at most 22 mammal species mentioned in the original article. The nearest non‐marine mammals of *Odobenus rosmarus divergens* , *Trichechus manatus latirostris* and the ancestor of *Tursiops truncatus* + *Orcinus orca* are *Canis familiaris* , *Loxodonta africana* and *Bos taurus* , respectively. For each protein alignment in the above two cases, sites with gaps and ambiguous amino acids were consistently excluded following the practice of previous studies (Thomas and Hahn 2015; Zou and Zhang 2015a). Alignments only containing one single site after filtering were discarded in subsequent analyses. For mangroves, a total of 5353 protein alignments, each containing three mangrove species ( *Avicennia marina* , *Rhizophora apiculata* , *Sonneratia alba* ), three non‐mangrove species ( *Sesamum indicum* , *Populus trichocarpa* , *Eucalyptus grandis* ) and one outgroup species *Oryza sativa* , were kindly provided by the authors of the original study (Xu et al. 2017). The distribution of the alignment lengths used in each case is shown in Figure S2.

### 2.2 Define the Convergence of Amino Acid Properties

We define convergence of amino acid properties (CAAP) as the same physicochemical property change happening in two or more independent species lineages (Figure 1b). According to a certain classification of the 20 amino acids by physicochemical properties, our definition of property convergence requires amino acid class changes on multiple focal branches leading to the same derived class. Consequently, unlike the conventional state convergence definition, sites experiencing convergent amino acid changes within the same property class are no longer considered convergent, whereas those experiencing physicochemical class changes and arriving at the same class, but not necessarily the same amino acid state, are defined as having property convergence. For example, if we denote two amino acid substitutions, A to B in one focal lineage and C to D in another focal lineage, as ABCD, then RHRH is not considered as property convergence, because R and H are both basic amino acids; meanwhile RDRD is property convergence because the basic R changes to the acidic aspartic acid D in both lineages. Notably, RDRE is property convergence but not state convergence by definition. We propose that focusing on property changes may both reduce the chance of observing neutral convergence by conservative changes and capture novel adaptive radical changes in sequence convergence analysis.

### 2.3 Group Amino Acids by Their Physicochemical Properties

To determine actual CAAP events in sequence data, a grouping of the 20 amino acids by physicochemical property similarity is needed. We employed four distinct group schemes (GS1–GS4) for categorising amino acids, so that different aspects and granularity levels of amino acid physicochemical properties were considered. Of these schemes, three (GS1, GS2, GS3) are coarse‐grained classifications, categorising 20 amino acids into six or seven classes based on their polarity and volume (Zhang 2000; Yang et al. 2010; Chaurasia and Dutheil 2022). The fourth scheme (GS4) adopts a more fine‐grained classification, only grouping amino acids with highly similar side chains together (Creighton 1996). To investigate the impact of grouping amino acids, we randomly shuffled the 20 amino acids between the six classes in GS1 to generate GS0: ‘GWDC’, ‘PM’, ‘K’, ‘IQLS’, ‘EATVYF’, ‘NHR’. We denote the scenario in which no grouping is applied as the ungrouped scheme (US). Details of each grouping scheme are shown in Figure 1c.

### 2.4 Detect Property Convergence

For each of the three organismal convergence cases we investigated in this study, the original method for detecting amino acid state convergence was first applied to the data for replicability, and then modified to detect property convergence for comparison. In general, to modify the original methods for property convergence detection, we recoded the amino acid states to their class indices according to each GS, that is, treating amino acids within the same class as having identical ‘state’. For the echolocating mammals, we replicated the R statistics (Zou and Zhang 2015a), which may indicate putative adaptive convergent sites based on the ratio of observed number of convergence (O) versus neutral expectation (E) under the gene model, that is, with amino acid frequencies estimated from the gene alignment sequences (Figure S3). To count O between a pair of branches, we first performed ancestral sequence reconstruction (ASR) using PAML 4.9j (Yang 2007) based on the LG substitution model. The reconstructed ancestral sequences with the largest site‐wise marginal likelihood at each node of the phylogeny were then compared accordingly, so as to identify amino acid changes at each site on each branch. We then identified sites with convergent patterns between focal branches in the sequence. To calculate E between a pair of branches, we first constructed the substitution matrix based on an empirical exchangeability matrix (e.g., from the LG matrix) and gene‐specific amino acid equilibrium frequencies. Other evolutionary parameters (i.e., rates, branch length) were inferred by PAML. Next, we calculated the probabilities of every type of simultaneous amino acid substitutions on both branches. We thereby identified all types of events defined as convergence and summed the probabilities up to obtain the expected number of convergence E for each site. Treating the rare convergent events as following a Poisson process, the E for the whole gene was derived by summing the site‐wise E up along the sequence. For testing CAAP under GS0‐4, we likewise computed the observed (O) and expected (E) number of convergent sites in the same manner as in the original study. R was calculated as O/E for each gene as in the original study, representing a relative abundance level of convergence. If an R value is significantly > 1, meaning the O exceeds the E, the gene is regarded as having experienced putatively adaptive convergence. We used the Poisson test to examine whether O > E, that is, whether *R* > 1, and set a cutoff of *p* < 0.05. Since the significant genes would go through further filtering such as functional enrichment analysis, no multiple‐testing correction was conducted at this stage.

To verify that O equals E under neutral evolution, we simulated 1000 gene alignments, each containing 500 amino acid sites in 36 species, by Markov process, following previous methods (Zou and Zhang 2015a). The simulation used exchangeabilities and equilibrium frequencies of the LG empirical matrix. A tree topology was randomly generated and used for all alignments, with branch lengths set to 0.1 for all branches. Two branches were manually chosen as the focal branch pair. The root sequences were randomly generated by sampling from the LG equilibrium frequencies, and the rates for each site in the alignment were drawn from a gamma distribution with a shape parameter equal to 2.

To investigate if the choice of different substitution models in ASR may significantly alter the analysis results, we changed the LG model used in the main analysis to the JTT model or the WAG model, and repeated the step of counting O values in 50 randomly sampled genes with O = 0 and another 50 randomly sampled genes with O > 0. The O values under different ASR substitution models were then tested for between‐model differences by Friedman test.

In the case of marine mammals, the authors determined convergent sites by comparing the amino acid states of the focal species and the most recent common ancestor (MRCA) of the focal species and their nearest sister species (Foote et al. 2015). We followed the same procedure, but recoded the amino acid states to property classes under GS0‐4 in the analyses. In the CCS method for convergence of woody mangroves, the authors first retained only conserved sites by requiring identical site states in the outgroup and all non‐mangrove species, and then looked for convergent sites with at least two mangrove species having the same amino acid state distinct from the non‐mangroves (Xu et al. 2017). To detect property convergence by CCS, we also retained only conserved sites by requiring identical amino acid states in the outgroup and all non‐mangrove species, and then looked for property convergent sites with at least two mangrove species exhibiting the same amino acid property class, which is different from the class in the outgroup and all non‐mangrove species.

### 2.5 Conduct Negative Control Analysis in Each Case

To validate that the genes with property convergence are not found by chance in each case, we designed negative control analyses to exclude possible false positive findings. For the case of echolocating mammals, in each of the two focal branch pairs (BP1 and BP2), we replaced the basal branch of the toothed whales with the nearest sister branch, which is the basal branch of the Bovidae (MRCA of *Bos taurus* , *Bos indicus* , *Bos mutus* , *Bison bison* , *Capra hircus* , *Ovis aries* , *Bubalus bubalis* and *Pantholops hodgsonii* ), as one focal branch to be compared with the echolocating bats. Analyses based on this replacement served as a negative control for detecting sites or genes that may be associated with the adaptive convergent evolution of echolocation.

For marine mammals, we followed the original paper (Foote et al. 2015) to use the corresponding terrestrial sister taxa ( *Bos taurus* , *Canis familiaris* , *Loxodonta africana* ) as a negative control. For woody mangroves, the negative control was derived by first retaining only conserved sites with identical amino acid states in the outgroup and all mangrove species. Then convergent sites among these conserved sites were identified, requiring at least two non‐mangrove species sharing the same amino acid states or properties distinct from those of the outgroup and the mangroves.

### 2.6 Explore Functional Correlation of Candidate Genes

To investigate the relation between genes with CAAP and the adaptive functions in respective ecological environments, Gene Ontology (GO) enrichment analysis and KEGG pathway enrichment analysis were conducted using the R package *clusterProfiler* (Wu et al. 2021). For GO enrichment in echolocating mammals, marine mammals and woody plants, we input gene Ensembl IDs and respectively used annotation from the *org.Hs.eg.db* (Carlson and Falcon 2019) package for mammals and annotation from the RAP‐DB (Sakai et al. 2013) and the OryzaBase (Kurata and Yamazaki 2006) for plants as reference. Enrichment of GO biological process, molecular function and cellular component terms was tested for gene sets in all three cases. In Table S4, repetitive terms found in *R* > 1 were ignored for clarity and similar terms found by three gene sets under the same GS and BP were represented by the term with the largest number of significantly enriched genes. KEGG pathway enrichment analyses were conducted for woody plants as in the original study. The Benjamini‐Hochberg method was used to correct the *p* values for multiple testing (Benjamini and Hochberg 1995). We focused on the genes assigned to significant terms with *p* < 0.01 in echolocating mammals, and with a false discovery rate (FDR) < 0.05 in marine mammals and woody plants for enrichment analysis, unless no such term exists. Functional relations of obtained individual genes with the focal organismal adaptive convergence (echolocation, marine‐dwelling, or mangrove lifestyle) were also investigated by manual literature research with relevant keywords.

### 2.7 Conduct PCOC Analysis and Positive Selection Test

The PCOC (Profile Change with One Change) method (Rey et al. 2018) was conducted in the case of echolocating mammals as an existing convergence detection method to be compared with our approach. It integrates two probabilistic submodels, the Profile Change (PC) model and the One Change (OC) model. The PC model detects shifts in amino acid frequency distribution across convergent branches, and the OC model enforces at least one substitution on focal branches. The PCOC calculates site‐specific posterior probabilities based on an empirical Bayesian framework by contrasting the likelihood of a null model (uniform ancestral profile across all branches) with that of a convergent model (divergent profiles on focal branches). A 0.99 posterior probability threshold was used, as it was validated through simulations and balances specificity and sensitivity to detect adaptive convergence in the presence of neutral noise, according to the original study.

To explore whether the genes with property convergence have experienced adaptation in the case of echolocating mammals, positive selection tests were conducted using the *codeml* program in PAML v4.9j (Yang 2007). Specifically, we aimed to detect whether certain sites in the genes are under positive selection on focal branches. To achieve this, we conducted a branch‐site test in PAML, assigning the focal branches with putative function convergence in each case as foreground branches. Two likelihood inferences were conducted based on the corresponding *codeml* control file of H0 (CodonFreq = 2, aaDist = 0, model = 2, NSsites = 2, fix_kappa = 0, fix_omega = 1, omega = 1) and H1 (CodonFreq = 2, aaDist = 0, model = 2, NSsites = 2, fix_kappa = 0, fix_omega = 0, omega = 2). The *p* value for rejecting the null (no positive selection) was obtained by the likelihood ratio test comparing H1 to H0. We used the Benjamini‐Hochberg method (Benjamini and Hochberg 1995) to correct for the multiple testing within the gene set of each GS or combination of GS and BP, and the FDR cutoff was 0.05.

## 3 Results

### 3.1 Detection of CAAP in Echolocating Mammals

We first investigated the convergent emergence of echolocation in mammals, involving the echolocating bats and toothed whales. Based on 8925 genes totalling 4,669,357 sites in 116 mammalian species (outgroup: *Ornithorhynchus anatinus* ), we followed Zou and Zhang (2015a) to calculate the metric R for each gene, as the ratio of the observed number of convergent sites (O) to the expectation under neutral substitution models (E). The calculation was conducted for two pairs of focal branches (BP1 and BP2) and modified to implement six different amino acid convergence schemes, that is, US and GS0‐4. To check if there are CAAP events, and particularly if there are additional instances of property convergence neglected by the original state convergence methods, we focused on four gene sets of interest: (1) genes with observed significantly more CAAP than neutral expectation, that is, *R* > 1 (*p* < 0.05); (2) genes with observed convergence under each scheme, that is, O > 0; (3) genes with observed convergence deviating more from the neutral expectation when amino acids are grouped, that is, R(GS) > R(US) for each GS; (4) genes with more observed convergence when amino acids are grouped, that is, O(GS) > O(US) for each GS. For simplicity, all genes with *R* > 1 mentioned hereafter satisfied the significance cutoff of *p* < 0.05.

We found that many genes show convergence in excess of the expectations (*R* > 1) in both US and GSs, and meanwhile each GS detects certain numbers of genes with O > 0, O(GS) > O(US) and R(GS) > R(US) (Table S1). We found 197, 127, 171, 173, 131 and 180 genes with *R* > 1 respectively in US and GS0‐4 under BP1, whereas for BP2, the numbers were 154, 108, 132, 130, 95 and 133 (red bars in Figure 2a). Notably, despite the highest number of *R* > 1 genes being consistently identified under the US across both BP1 and BP2, each GS still revealed additional genes with *R* > 1 that were not found under US (Figure S4a,b). Similarly, for genes with O > 0 (blue bars in Figure 2a), whereas the US scheme exhibited the most unique genes (BP1) or the highest number of genes (BP2), genes unique to the GSs were found as well (Figure S4c,d). Hence, we found a significant number of CAAPs in many genes, and moreover observed genes with CAAPs but not traditionally defined amino acid state convergence.

Next, we investigated whether the observed CAAPs were only the trivial effect of grouping amino acids but not helpful for evaluating the adaptive convergence of their physicochemical properties. Intriguingly, the random grouping scheme GS0 detected the highest number of genes with O > 0 across all GSs, which is meanwhile fewer than those found under US. However, GS0 identified the fewest genes with *R* > 1 (Figure 2a). The same pattern was true for genes with O(GS) > O(US) and genes with R(GS) > R(US). The numbers of genes with R(GS) > R(US) (yellow bars in Figure 2a) are generally higher than those of genes with O(GS) > O(US) (green bars in Figure 2a) because higher R under GS than R under US can result not only from an increase in observed convergence events O but also from an unchanged O and a decreasing convergence expectation E. Hence, for a gene, R(GS) > R(US) can indicate either more property convergent events observed or fewer property convergent events expected than state convergence. Although more genes with O(GS) > O(US) were observed under GS0 than under any of the property‐related GSs, GS1‐4 detected two to four times as many genes with R(GS) > R(US) as GS0 (Figure 2a). This could be explained by the higher exchange rates between the random classes in GS0, due to substitutions between amino acids with similar properties that were stochastically assigned into different classes, thus driving both O and E high under GS0. In contrast, amino acid grouping alone cannot explain the high R values we observed under GS1‐4. Hence, additional CAAP genes, putatively with adaptive convergence unexpected by neutral evolution, can be detected only when amino acids are grouped by actual physicochemical properties rather than random labels. The numbers of genes in different gene sets vary among GS1‐4 and branch pairs, suggesting that the choice of amino acid grouping scheme and phylogenetic hypothesis may impact the detection of convergence. We used the *R* > 1 sets for each GS and BP in the following analysis because the statistical significance cutoff can reduce the noise introduced by non‐adaptive convergent substitutions, according to the above findings.

To further benchmark the CAAP approach, we conducted three more analyses. First, we simulated sequence evolution in a random phylogeny under the neutral substitution model and then calculated R values between two lineages as well as derived the *P* values. We confirmed that in these negative control alignments, the observed convergence O equals the expectation E, that is, *R* = 1 (*p* > 0.4, Table S2). This validated that our R calculation and test were unbiased. Second, we investigated the potential impact of ASR on detecting CAAP events. Based on ASR under the JTT model or the WAG model, we found that no CAAP was observed (O = 0) in either BP1 or BP2 under all GSs in all 50 randomly sampled genes with O = 0 under the LG model. Meanwhile, for both branch pairs (BP1 and BP2) of 50 genes with O > 0 under the LG model, thus totaling 100 (50 × 2) cases, O = 0 was observed under one or more GSs in only seven (JTT) and four (WAG) cases. Furthermore, under all GSs, the statistical differences between total O values under LG, JTT and WAG models were insignificant (*p* > 0.2, Friedman test). Hence, the CAAP results were robust to model choice in ASR.

To benchmark the finding under the CAAP concept by existing methodologies, we compared our results with those of the PCOC (Profile Change with One Change) method (Rey et al. 2018). The PCOC method identified 617 and 641 PCOC genes for BP1 and BP2, respectively, nearly four times the number of genes with *R* > 1. For seven previously reported hearing genes (*SLC26A5*, *PJVK*, *OTOF*, *CDH23*, *TMC1*, *KCNQ4*, *PCDH15*) associated with echolocation in bats and toothed whales (Parker et al. 2013), PCOC reported significance in four genes, whereas the same number of genes showed significance with *R* > 1. The genes *OTOF* and *PCDH15* were detected only by PCOC, whereas the genes *SLC26A5* and *PJVK* were identified exclusively using our R metric under the CAAP concept (Table S3). Notably, the PCOC method detects convergent shifts of amino acid preferences in the focal lineages, without explicit modelling of physicochemical similarity between amino acids. Hence, our CAAP approach and the PCOC method do not necessarily detect the same signals in the sequence data.

### 3.2 Functional Enrichment of Genes With CAAP in Echolocating Mammals

To explore and understand the functions of genes under putative adaptive convergence, we conducted Gene Ontology (GO) enrichment analysis for genes with *R* > 1 in US and GS0‐4 in either BP1 or BP2, against a background of 8925 genes. We found a total of 93 GO terms enriched under GS1‐4 and two BP combinations, with a significance cutoff of *p* value < 0.01. We compared the genes involved in the enriched GO terms between US and GS1‐4, and found that while there were some overlaps between the US and the GSs, different GSs did uncover extra genes (Figure S5). To focus on the functions found uniquely by our CAAP gene enrichment analyses, we first removed redundant GO terms and then excluded the significantly enriched GO terms found by US and GS0 from the total GO term sets under respective BPs (Table S4). 51 CAAP‐specific GO terms were retained after this step (Table S4).

As a negative control, we repeated the CAAP analysis after changing one focal lineage from the toothed whales to the bovids in the R calculations, and found a relatively larger number of genes with *R* > 1 than in the original analysis (Table S6). This observation of excessive convergence in sister taxa of focal species is consistent with the previous studies (Thomas and Hahn 2015; Zou and Zhang 2015b). For these CAAP genes, we also conducted GO enrichment analysis and found that none of the resulting GO terms overlapped with the previously derived 51 CAAP‐specific terms (Table S7). This finding supported that the CAAP‐specific terms and corresponding involved genes may indeed be associated with convergent functional adaptation of echolocating mammals.

Next, we explored the functions of these genes involved in the remaining 51 CAAP‐specific terms by literature research and found that a substantial proportion of all 109 involved genes shows functions associated with hearing or vision in bats and toothed whales (Table S8). For example, the gene *PIEZO2* showed no state convergence but one property convergent site. The site harbours mostly glycine in echolocating bats and alanine in toothed whales, both of which are small amino acids, whereas other mammal species use the relatively larger valine (Figure 2b). Intriguingly, *PIEZO2* encodes a mechanosensitive ion channel essential for ultrasonic hearing via cochlear outer hair cells in mice (Li et al. 2021). Similarly, property convergence events such as M to I/L and V to I/L were observed in the genes *SCN11A* and *CHRNB2*; both genes have also been associated with hearing (Figure 2b and Table S8).

### 3.3 Genes With CAAP Are Under Positive Selection in Echolocating Mammals

We then did branch‐site tests of positive selection in genes with *R* > 1 (*p* value < 0.05) under US and GS1‐4. In total, 317 unique genes (union set of *R* > 1 genes under all schemes) and 235 unique genes were tested on BP1 and BP2 respectively. We found 12 genes (*CCDC92*, *CDH19*, *TEX14*, *CPA3*, *HPGD*, *WNK3*, *JPH3*, *VCAM1*, *CHAT*, *ELK3*, *CYSTM1*, *PRR14L*) exhibiting significance in the branch‐site test using the echolocating bats and toothed whales as focal lineages (FDR < 0.05, Table 1 and Table S9). Regarding physicochemical property convergence, 11 of the 12 genes contained CAAP sites, except *VCAM1*. Moreover, both *WNK3* and *JPH3* possessed only one CAAP site but no convergent site under the US (Table 1). In contrast, for the negative control branch pair of echolocating bats and bovids, 10 out of the 12 genes showed no convergent site, except for *TEX14* (2–3 detected sites under US and GS1‐4) and *HPGD* (one site under US and GS4) (Table S10). Based on literature research, most of the 12 genes are functionally relevant to hearing (Table S9). For example, *JPH3* exhibits an exclusive property convergence event of TTAI under GS4 in BP2, and has been reported to show increased expression during the maturation of mouse primary auditory cortex (Hackett et al. 2015). *WNK3* has an exclusive property convergence event of PPRH under GS1/3/4 in BP2, and can dynamically regulate balance between Na‐K‐2Cl cotransporters (NKCCs) and K‐Cl cotransporters (KCCs) activities (Kahle et al. 2010). Studies have found that inactivation of NKCCs was associated with deafness (Delpire et al. 1999), and KCC4 expression can be located in the inner ear and loss of KCC3/KCC4 caused deafness (Boettger et al. 2002; Boettger et al. 2003). *TEX14* exhibited two exclusive property convergence events of VVIL and TTIP among all seven under GS2 in BP1. *TEX14* has been reported to show 14.5 times higher expression in the cochlea of mice with impending hearing loss than that of wildtype mice, potentially mediating cellular protrusion formation and cytoskeletal organisation (Dufek et al. 2020). Besides, the cadherin *CDH19* is expressed in the cochlea and significantly down‐regulated in a middle ear disease (Klenke et al. 2012; Lin et al. 2012); *PRR14L* is in a deletion that can cause human hearing loss (Trizuljak et al. 2023); *CHAT* encodes an enzyme responsible for synthesising the neurotransmitter acetylcholine, which is significantly decreased in the auditory cortex of mutated mice (Hacohen‐Kleiman et al. 2019). The above findings suggest that these genes with CAAP in echolocating mammals are likely associated with adaptive organismal convergence for hearing‐related functions.

**TABLE 1:** Genes with *R* > 1 (*p* value < 0.05) and under significant positive selection for US and all GSs, and both branch pairs in echolocating mammals.

Gene name	Branch pair	Number of convergent sites					FDR
		US	GS1	GS2	GS3	GS4	
*CCDC92*	BP1	2	—^a^	—	0	2	5.13 × 10^−3^
*CDH19*	BP1	2	—	2	—	2	2.64 × 10^−3^
*TEX14*	BP1	5	6	7	4	5	2.77 × 10^−3^
*CPA3*	BP1	3	—	—	—	—	2.74 × 10^−2^
*HPGD*	BP1	1	0	0	0	1	8.75 × 10^−3^
*WNK3*	BP2	0	—	0	1	—	1.89 × 10^−4^
*JPH3*	BP2	0	0	0	0	1	3.12 × 10^−2^
*VCAM1*	BP2	1	0	0	0	0	4.33 × 10^−2^
*CHAT*	BP2	1	1	1	1	1	1.39 × 10^−2^
*ELK3*	BP2	1	1	1	1	1	2.17 × 10^−2^
*CYSTM1*	BP2	1	1	1	0	1	6.93 × 10^−6^
*PRR14L*	BP2	1	1	1	1	1	2.33 × 10^−3^

^a^ A dash (—) indicated that the *R* value for the gene was not significant under the respective grouping scheme.

### 3.4 Genes With CAAP in Marine Mammals and Woody Mangroves Are Related to Putative Adaptive Functions More Frequently Than Genes With Conventional State Convergence

**FIGURE 3:**
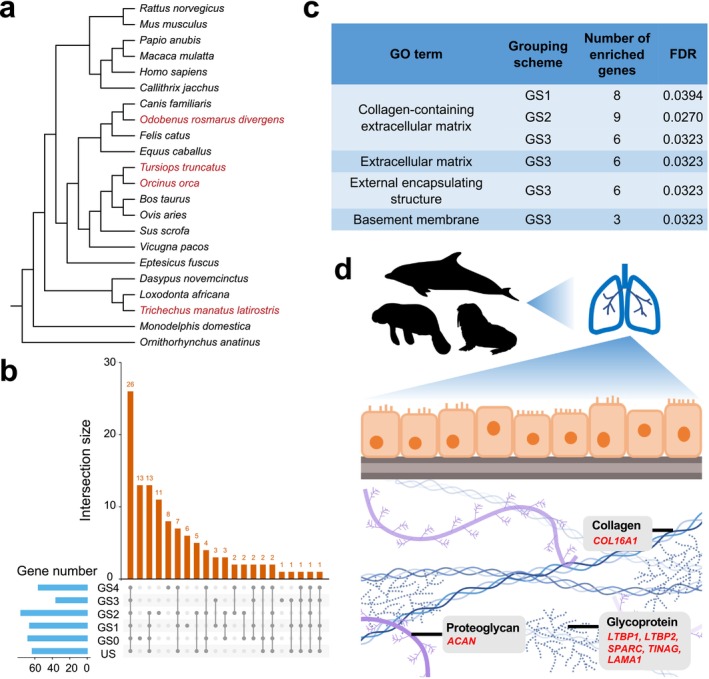
Genes exhibiting property convergence are functionally related to organismal convergence in marine mammals. (a) Species tree of 22 mammalian species. Focal marine mammals are highlighted in red. (b) The number of genes with detected convergent sites in all three lineages in each intersection of US and GS0‐4. (c) GO enrichment results of the gene sets with convergent sites detected in all three lineages under each GS in marine mammals. GO terms with FDR < 0.05 were shown. (d) Diagram of mammalian extracellular matrix components, according to Soles et al. (2023) and Wijsman et al. (2021). Convergent genes associated with the formation of the lung core matrisome are shown in red. Only collagens, proteoglycans and glycoproteins are illustrated. Silhouettes of mammal species were retrieved from phylopic.org.

To verify the potential contribution of amino acid property convergence to organismal adaptive convergence processes in other taxonomic groups, we further investigated the cases of marine mammals and woody mangroves. Mammals including whales, pinnipeds and manatees may have adapted to marine life by distinctive traits across multiple systems, for example, the respiratory and the musculoskeletal systems. Focusing on the walrus ( *Odobenus rosmarus* ), the bottlenose dolphin ( *Tursiops truncatus* ), the killer whale ( *Orcinus orca* ) and the manatee ( *Trichechus manatus latirostris* ), Foote et al. (2015) detected amino acid state convergence (denoted as ‘parallel’ site in the publication) in all three lineages or two of the three lineages (Figure 3a). We replicated their results of state convergence in 12,727 orthologous genes of 22 mammalian species using the original method (US, Figure S6), and then counted property convergence by GS1‐4. Focusing on three‐lineage convergence events, we found 38 genes with exclusive property convergence in addition to 63 genes with both property and state convergence (Figure 3b). Applying a *P* value cutoff of 0.05, as in the original study, two of the 38 genes exhibited positive selection signals, whose functions are both putatively associated with marine life adaptation (Table S11).

GO enrichment analyses of the 63 genes found under US resulted in no significant term (Figure S7). In contrast, the term ‘collagen‐containing extracellular matrix’ was significantly enriched among property convergence genes under GS1‐3, totaling 12 genes, alongside other highly repetitive terms under GS3 (FDR < 0.05, Figure 3c). Among the 12 genes, *SERPINC1* has been reported in a previous analysis of marine mammals (Foote et al. 2015) and five genes exhibit exclusive property convergence sites. These genes do not overlap with previously reported genes with accelerated evolutionary rates in the three marine lineages (Chikina et al. 2016), probably because convergent acceleration of sequence evolution does not necessarily lead to similarity of the sequence per se. Intriguingly, among the 12 genes, *ACAN*, *LTBP1*, *LTBP2*, *COL16A1*, *TINAG*, *SPARC* and *LAMA1* are all components of the core matrisome in lung extracellular matrix (ECM) (Figure 3d), whereas *ITIH1*, *S100A8* and *MMP2* are also associated with matrisome (Zhou et al. 2018) (Table S12). As structural support for cells, ECM is important for lung development, homeostasis and injury repair (Zhou et al. 2018). Hence, our results suggest prevalent adaptive convergence for underwater respiration in lung ECM of marine mammals, which was not reported by previous analyses focusing on state convergence.

As a negative control for the above results, we further detected the convergence in the three sister terrestrial taxa and found a larger number of genes with CAAP than in the marine mammals. Only two GO terms were significantly enriched, and they were not associated with aquatic adaptation (Table S13). The remarkable level of convergence found in the terrestrial sister taxa was consistent with the previous study (Foote et al. 2015). Under the random grouping GS0, we found 68 convergent genes, which include the highest number of unique genes not found under other GSs. In contrast, GO enrichment analysis for these genes found under GS0 derived only two significant GO terms, and only 3 out of 15 involved genes were related to putatively adaptive function (Table S14). These results further supported the potential relevance of the CAAPs found in marine mammals to specific adaptation.

**FIGURE 4:**
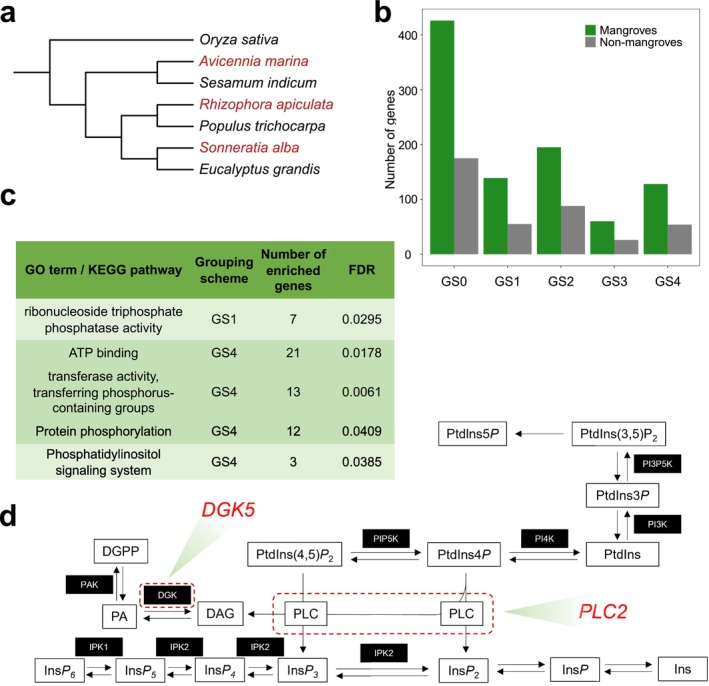
Genes exhibiting property convergence are functionally related to organismal convergence in mangroves. (a) Species tree of the seven species used for analysis. Mangrove species are highlighted in red. (b) The numbers of genes showing an increased number of convergent sites in mangroves and non‐mangroves under GS0‐4. (c) GO and KEGG pathway enrichment results of the gene sets with increased number of convergent sites in mangroves under GS1 and GS4. GO terms and KEGG pathways with FDR < 0.05 were shown. For simplicity, redundant GO terms are omitted here and can be referred to in Table S16. (d) Diagram showing genes involved in phosphatidylinositol signalling and metabolism in plants. Core enzymes encoded by genes with property convergence are outlined with red dashed boxes; respective gene names are enlarged in red.

Next, we investigated the markedly different case of mangroves, that is, specialised woody plants adapted to unique coastal environments with high salinity, extreme tidal variations, high temperature and muddy anaerobic soils (Xu et al. 2017). A previous study by Xu et al. (2017) looked for convergence at conservative sites (CCS) in three independent mangrove lineages, using three non‐mangrove sister lineages and the outgroup rice as background (Figure 4a). Based on 5353 orthologous genes of the seven species, we detected the same number of genes with state convergence (US) for mangroves and non‐mangroves as the original study and obtained comparable KEGG pathway enrichment results (Table S15 and Figure S8b). By modifying the CCS method to detect property convergence, we found generally more genes with an increased number of convergent sites under GS0‐4 in mangroves than in non‐mangroves (Figure 4b). Genes found under GS1 and GS4 in mangroves were significantly enriched in the GO terms of ATP binding, transferase activity or protein phosphorylation, and the KEGG pathway ‘Phosphatidylinositol signaling system’ (FDR < 0.05, Figure 4c and Table S16), whereas no GO term or KEGG pathway was significantly enriched under US (Figure S8a,b) or other GSs. Similar to the observations in the previous two cases, whereas more CAAP genes were detected in mangroves and non‐mangroves under GS0 than under GS1‐4, no significant GO terms or KEGG pathway were enriched for this random grouping scheme. As a negative control, we selected genes with an increased number of convergent sites in the three non‐mangroves. Consequently, no GO term or KEGG pathway was enriched under GS1 or GS4 (Figure S9). Protein phosphorylation is important in responding to abiotic stresses including salt, heat and wind stress in plants (Ichimura et al. 2000) and phosphatidylinositol (PI) signalling is induced and functions during osmotic stress (Di Paolo and De Camilli 2006). Genes like *WNK1*, *CDKE1*, *DGK5* and *PLC2* may play key roles in such stress responses (Table S17, Figure 4c, adapted from Munnik and Vermeer (2010)). Hence, our investigation into property convergence events in marine mammals and woody mangroves has uncovered additional genes and pathways putatively underlying adaptive functional convergence.

## 4 Discussion

In summary, we asked in this study whether convergence of amino acid properties (CAAP) can be the molecular basis of adaptive functional convergence at the organismal level. We developed pipelines to detect CAAP by modifying existing methods, and applied the pipelines to three different cases of organismal convergence, namely echolocating mammals, marine mammals and woody mangroves. Functions and pathways related to the organismal adaptation were found statistically enriched among genes with CAAP, and fewer adaptation‐related terms were found among genes with amino acid state convergence in marine mammals and mangroves. We also discovered multiple CAAP genes under positive selection in focal convergent lineages of echolocating species. Our findings supported the conclusion that convergence of amino acid physicochemical properties may underlie adaptive functional convergence, and demonstrated that the CAAP concept is applicable in various biological cases and different methods. We provide the source code of the modified R and CCS analysis pipelines for detecting property convergence (available at https://github.com/shanschen33/CAAP).

To account for different aspects and granularity of physicochemical properties of amino acids, we used four different grouping schemes. The GS1‐3 grouped amino acids into six or seven classes based on their polarity and volume. Although GS1 and GS2 strictly group amino acids based on polarity and volume, GS3 has a group of amino acids, A, G, P, S and T, with ‘neutral and small’ properties. This group mixes the polar and nonpolar small amino acids, which may therefore lead to the decrease of convergence in GS3. For example, a site with a nonpolar A to polar T substitution in both focal branches would not be considered a property convergence site in GS3. In addition, the GS4 defines 12 classes, considering the more complex physicochemical properties (e.g., basic, acidic, hydroxylic) of the amino acids. For example, the enriched gene *LOC_Os03g48300* (*OsVIP2*) is a downstream gene for Ins*P*_6_ synthesis and harbours a CAAP (basic K to acidic D or E) at site 617 for mangroves (Table S17). This property convergence may be related to salty environment adaptation, as researchers have found that halophiles favour acidic over basic amino acid residue to enable intrinsically stable proteins to bind to water and salt (Jaenicke 1991). We note that such relevance is preliminary and warrants further exploration. Hence, in the current analysis practice, we suggest trying out different GSs to fully explore the putative functional‐related property convergence.

Focusing on CAAP rather than the conventional state convergence may both capture additional adaptation signals of radical sequence changes and reduce the noise of neutral conservative changes. Thus, CAAP may potentially be more suitable for detecting adaptive sequence convergence. Notably, there are several methods to detect profile shifts of amino acids, associating the shifts with directional selection, such as PCOC and Pelican (Tamuri et al. 2009; Rey et al. 2018; Duchemin et al. 2023). However, these methods do not explicitly model the physicochemical similarity and difference between individual amino acids. For example, preference changes between two acidic amino acids would contribute to profile change, but not CAAP. One potential caveat of our analysis method is that the definition of CAAP events is based on empirical, discrete categories of amino acids. Prospectively, the quantitative, continuous alterations of amino acid physicochemical properties, which have been revealed by existing evidence to form a universal component of sequence evolution (Tang et al. 2004; Chen, He, et al. 2019; Chen, Lan, et al. 2019; Zhang et al. 2024), could be integrated into CAAP detection. In fact, physicochemical properties are higher‐level features of the protein sequence than individual amino acid states. The molecular convergence underlying adaptive convergence of organismal functions can reside on various levels from site‐wise amino acid states to protein structure (Aminetzach et al. 2009) and gene expression regulation (Nagy et al. 2014). Such complexity calls for further exploration of the methodology of measuring convergence at various levels of sequence features, which is essential for our eventual comprehensive characterisation of mechanisms in adaptive organismal convergence.

## Author Contributions

Z.Z. supervised the study. Z.Z. and S.C. conceptualised the study and designed the methods. S.C. conducted all analyses and wrote the draft. Z.Z. and S.C. visualised the results and completed the writing by reviewing and editing.

## Conflicts of Interest

The authors declare no conflicts of interest.

## Supporting information


**Figure S1:** Species tree of 116 mammals used in the detection of property convergence.
**Figure S2:** Distribution of sequence length in each case.
**Figure S3:** Schematic overview of the CAAP gene identification workflow.
**Figure S4:** Overlap of convergent genes found under different schemes for echolocating mammals.
**Figure S5:** Overlap of enriched genes in significant GO terms found by genes with *R* > 1 in US and GS1‐4.
**Figure S6:** Number of convergent sites for each lineage combination, corresponding to Figure 2 in Foote et al. (2015).
**Figure S7:** GO enrichment result of the gene set with three‐lineage convergent sites under US in marine mammals.
**Figure S8:** Enrichment results of the convergent genes under US in mangroves.
**Figure S9:** Enrichment results of the gene sets with increased convergent sites under GS1 and GS4 in nonmangroves.
**Table S1:** Genes of R > 1, O > 0, O(GS) > O(US) and R(GS) > R(US) for each combination of BP and scheme.
**Table S2:** The O, E, R and Poisson test P values for US and all GSs in two lineages of the 1000 sequences alignments simulated under the neutral substitution models.
**Table S3:** Compare the results of genes with *R* > 1 and the PCOC model for seven previous genes associated with adaptive functions in echolocating mammals.

**Table S4:** All significant enriched GO terms for US and GS0 in echolocating mammals.
**Table S5:** 51 enriched GO terms for each combination of BPs and GSs for the three gene sets of *R* > 1.
**Table S6:** The number of genes with *R* > 1 between the echolocating bats and non‐echolocating bovids under the US and GS1‐4.
**Table S7:** All significant enriched GO terms for each combination of BPs and GSs in echolocating bats and non‐echolocating bovids.
**Table S8:** GO enrichment result of genes with *R* > 1 (*p* value < 0.05) in all combinations of GS1‐4 and BPs for echolocating mammals.
**Table S9:** Genes with *R* > 1 (*p* value < 0.05) in US and GS1‐4, and under positive selection in echolocating mammals.
**Table S10:** The number of sites showing convergence between echolocating bats and nonecholocating bovids, within genes under positive selection in echolocating mammals.
**Table S11:** Genes with exclusive property convergence and under positive selection in marine mammals.
**Table S12:** Genes with CAAP enriched in ‘collagen‐containing extracellular matrix’ for different GSs in marine mammals.
**Table S13:** The number of genes with CAAP and enriched GO terms in the terrestrial sister species of marine mammals.
**Table S14:** Non‐repetitive enriched GO terms and involved genes for GS0 in marine mammals.
**Table S15:** Number of convergent genes for mangroves and non‐mangrove and the result of KEGG (corresponding to the Fig. 2a in Xu et al. (2017)).
**Table S16:** All significantly enriched GO terms under different GS in mangroves.
**Table S17:** GO and KEGG pathway enrichment result of the gene sets with increased convergent sites in mangroves under GS1 and GS4.

## Data Availability

All data sources needed to evaluate the conclusions in the paper are present in the paper and the Supporting Information. We provide the source codes of the modified R calculation and the modified CCS method as analysis pipelines named CAAP (Convergence of Amino Acid Property), and the Python scripts are available at https://github.com/shanschen33/CAAP. Additional data and scripts related to this paper may be requested from the authors.
