# Supplementary material for: Detecting Convergence of Amino Acid Physicochemical Properties Underlying the Organismal Adaptive Convergent Evolution
Source: Mol Ecol Resour. 2025 Sep 26;25(8):e70052. doi: 10.1111/1755-0998.70052 (PMC12550464; doi:10.1111/1755-0998.70052)
Supplement: Supplementary file 1 — Figure S1: Species tree of 116 mammals used in the detection of property convergence. Figure S2: Distribution of sequence length in each case. Figure S3: Schematic overview of the CAAP gene identification workflow. Figure S4: Overlap of convergent genes found under different schemes for echolocating mammals. Figure S5: Overlap of enriched genes in significant GO terms found by genes with R > 1 in US and GS1‐4. Figure S6: Number of convergent sites for each lineage combination, corresponding to Figure 2 in Foote et al. (2015). Figure S7: GO enrichment result of the gene set with three‐lineage convergent sites under US in marine mammals. Figure S8: Enrichment results of the convergent genes under US in mangroves. Figure S9: Enrichment results of the gene sets with increased convergent sites under GS1 and GS4 in nonmangroves. Table S1: Genes of R > 1, O > 0, O(GS) > O(US) and R(GS) > R(US) for each combination of BP and scheme. Table S2: The O, E, R and Poisson test P values for US and all GSs in two lineages of the 1000 sequences alignments simulated under the neutral substitution models. Table S3: Compare the results of genes with R > 1 and the PCOC model for seven previous genes associated with adaptive functions in echolocating mammals. [file MEN-25-e70052-s002.pdf]

Supplementary Materials for

Detecting convergence of amino acid physicochemical property underlying the organismal adaptive convergent evolution

Shanshan Chen, Zhengting Zou*

*Corresponding author. Email: zouzhengting@ioz.ac.cn

This PDF file includes:

Figs. S1 to S9

Tables S2, S3, S6, S8-S17

Supplementary References

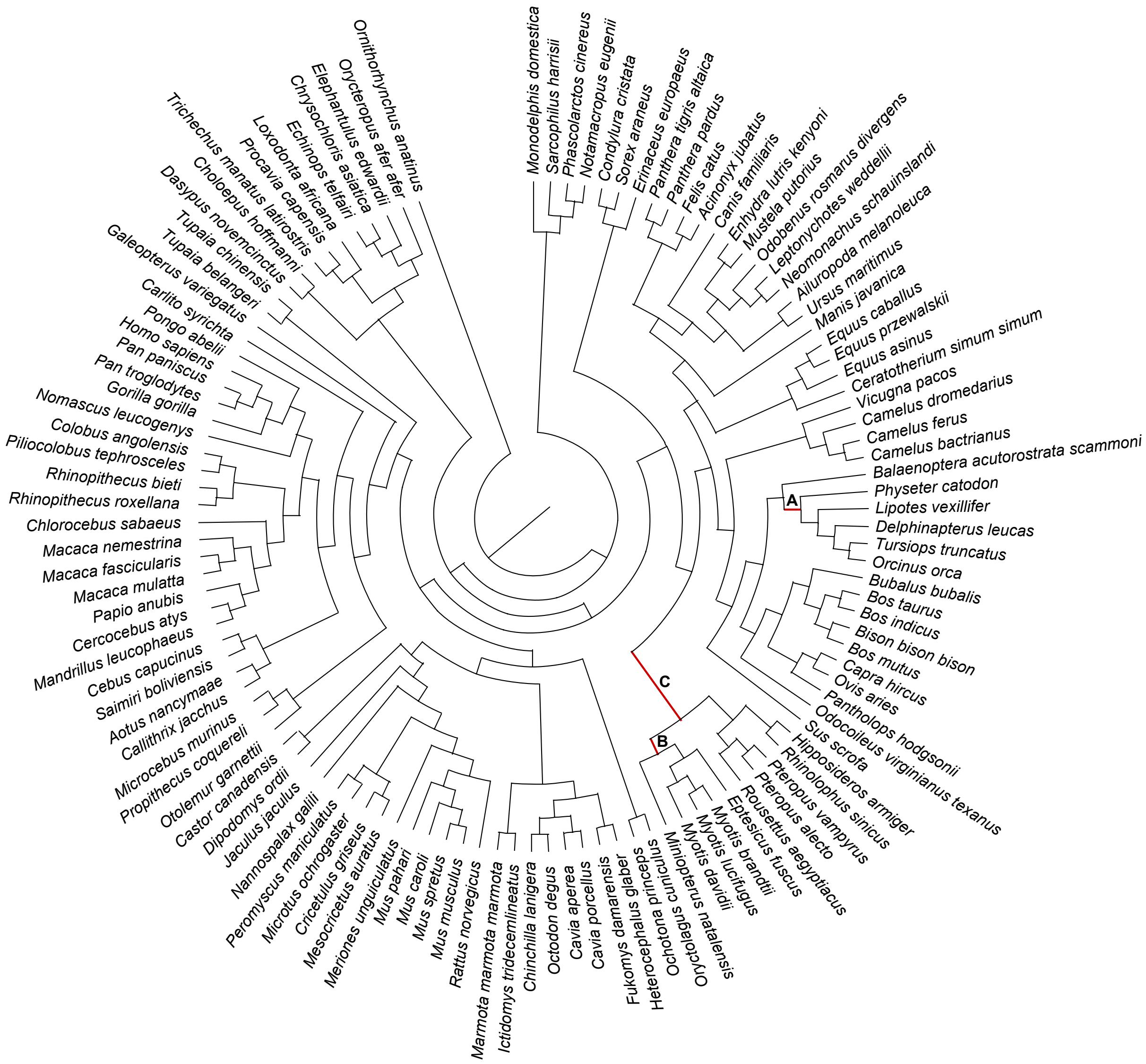

Fig. S1.

Species tree of 116 mammals used in the detection of property convergence.

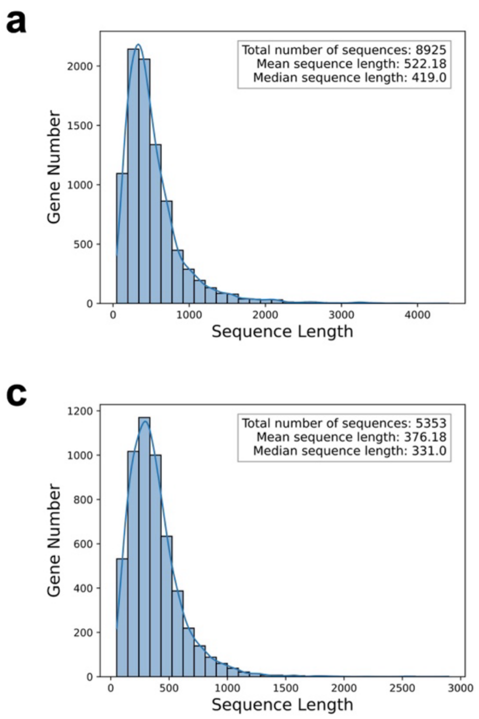

Fig. S2.

Distribution of sequence length in each case. (a) Sequence length distribution of genes used in echolocation case. (b) Sequence length distribution of genes used in marine mammal case. (c) Sequence length distribution of genes used in mangrove case.

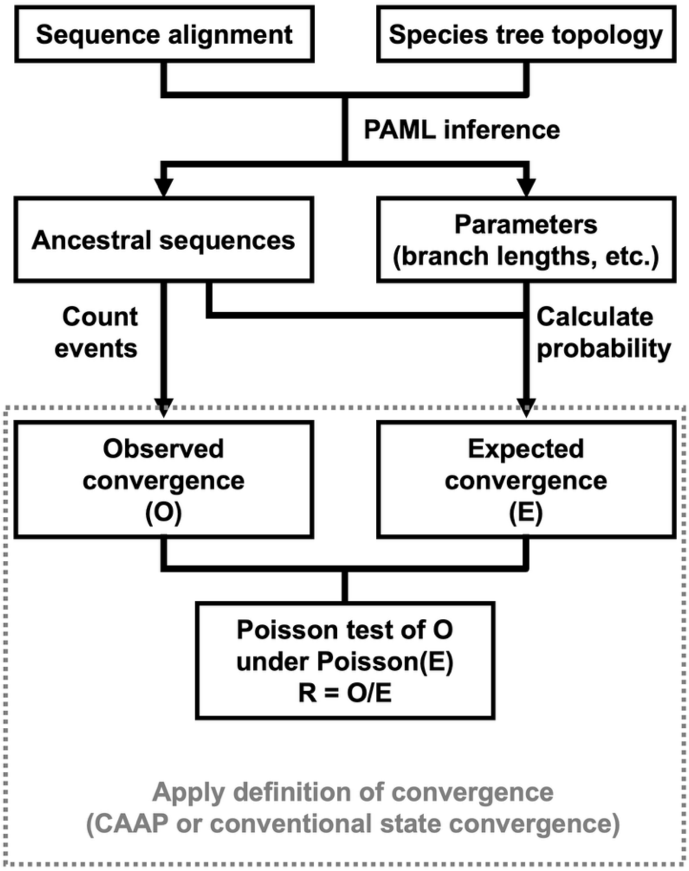

Fig. S3.

Schematic overview of the CAAP gene identification workflow.

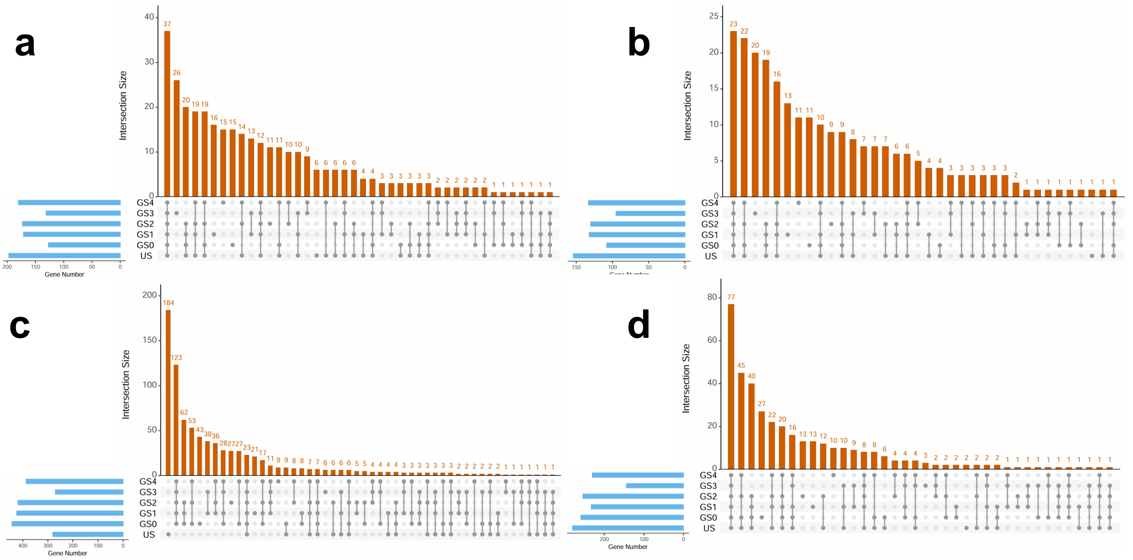

Fig. S4.

Overlap of convergent genes found under different schemes for echolocating mammals. (**a**) Venn diagram of genes with R > 1 between GSs and the US detected in BP1. (**b**) Venn diagram of genes with R > 1 between GSs and the US detected in BP2. (**c**) Venn diagram of genes with O > 0 between GSs detected in BP1. (**d**) Venn diagram of genes with O > 0 between GSs detected in BP2.

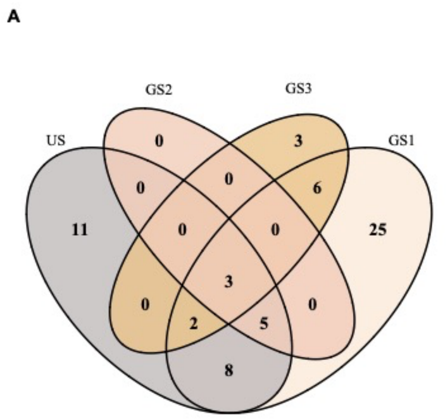

Fig. S5.

Overlap of enriched genes in significant GO terms found by genes with R > 1 in US and GS1-4. (**a**) Venn diagram of enriched genes in US and GS1-4 in BP1. (**b**) Venn diagram of enriched genes in US and GS1-4 in BP2.

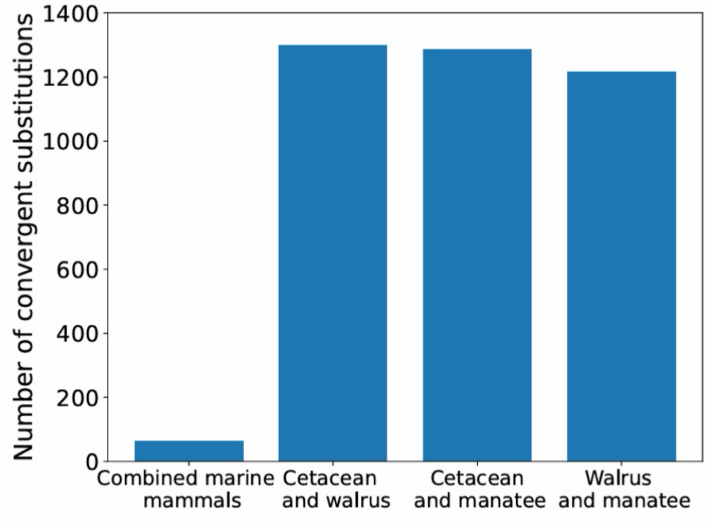

Fig. S6.

Number of convergent sites for each lineage combination, corresponding to Fig. 2 in Foote, et al. (2015).

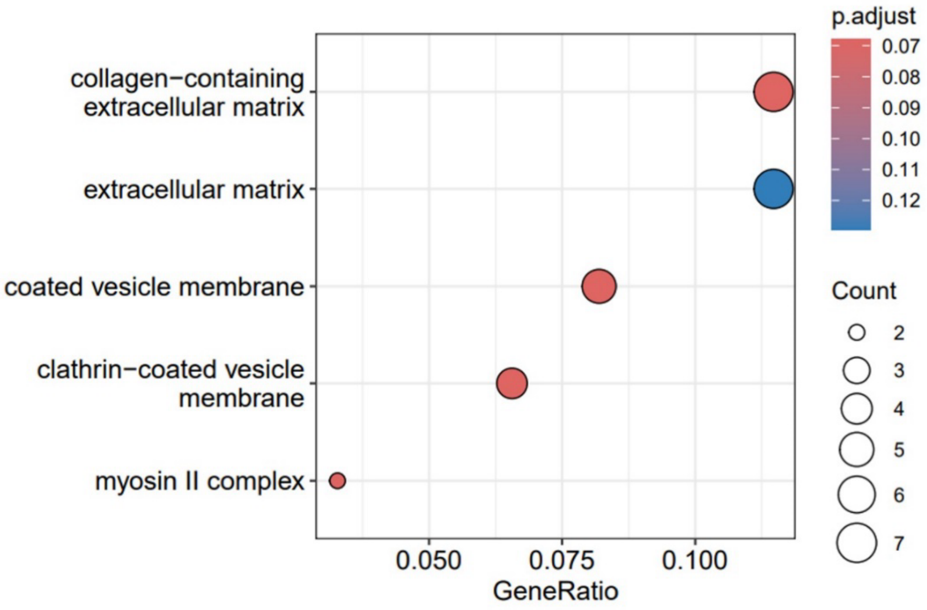

Fig. S7.

GO enrichment result of the gene set with three-lineage convergent sites under US in marine mammals.

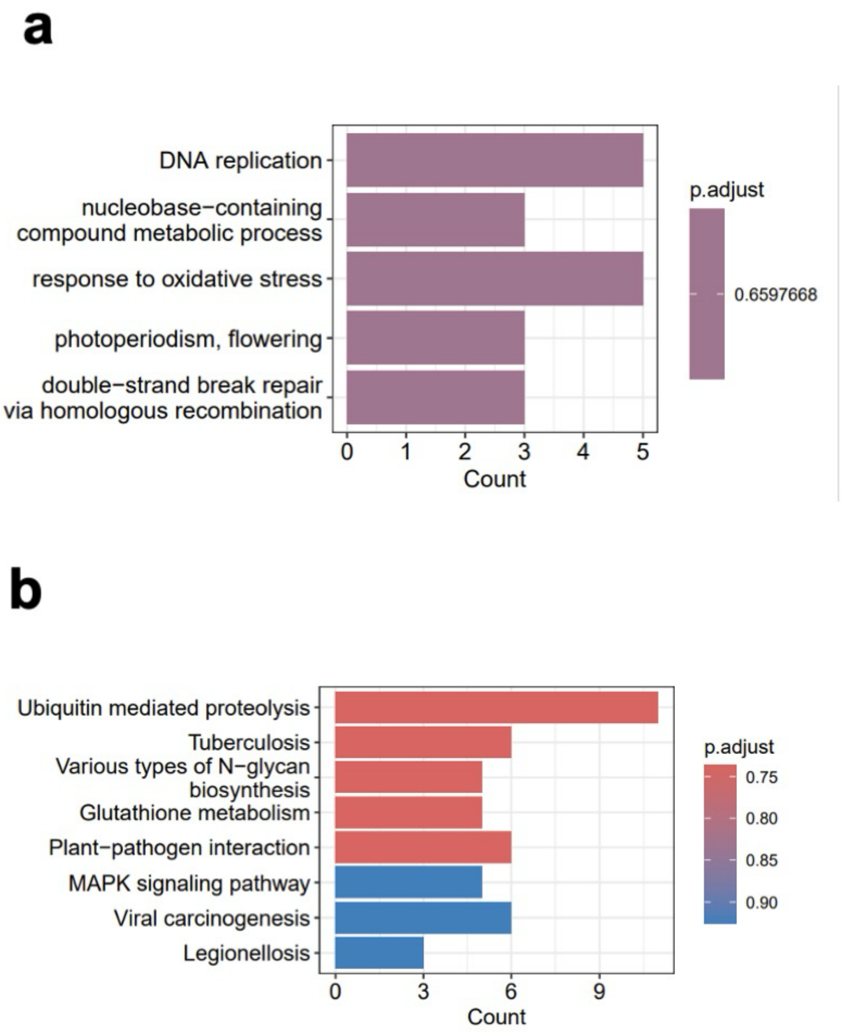

Fig. S8.

Enrichment results of the convergent genes under US in mangroves. (**a**) GO enrichment in mangroves under US. (**b**) KEGG enrichment in mangroves under US.

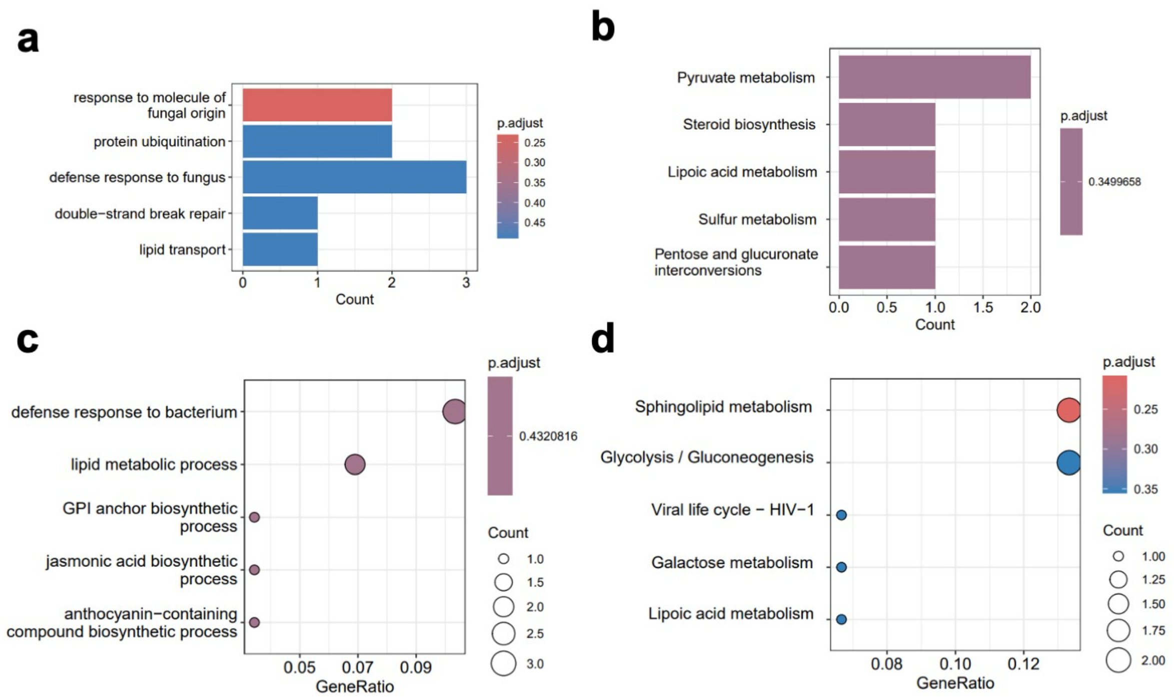

Fig. S9.

Enrichment results of the gene sets with increased convergent sites under GS1 and GS4 in non-mangroves. (**a**) GO enrichment in non-mangroves under GS1. (**b**) KEGG enrichment in non-mangroves under GS1. (**c**) GO enrichment in non-mangroves under GS4. (**d**) KEGG enrichment in non-mangroves under GS4.

Table S2. The O, E, R and Poisson test *P* values for US and all GSs in two lineages of the 1,000 sequences alignments simulated under the neutral substitution models.

Statistic	US	GS0	GS1	GS2	GS3	GS4
**O**	1788	2739	1584	1926	973	1425
**E**	1784.509	2717.753	1603.804	1946.654	999.1578	1411.927
**R**	1.002	1.008	0.988	0.989	0.974	1.009
***P* value**	0.934	0.680	0.635	0.650	0.420	0.719

Table S3. Compare the results of genes with R > 1 and the PCOC model for seven previous genes associated with adaptive functions in echolocating mammals. The values in US and GS0-4 represent the Poisson test *P* values. The *P* values < 0.05 in GS1-4 were highlighted in red. The '-' indicated no CAAP was found, and the Y / N corresponding to genes can / cannot be found by PCOC.

Gene	BP	US	GS0	GS1	GS2	GS3	GS4	PCOC
SLC26A5	BP1	-	-	-	-	-	-	N
	BP2	0.0575	0.0796	-	0.0628	-	0.0297	N
PJVK	BP1	0.0434	0.0567	-	-	-	0.0230	N
	BP2	0.0135	0.0176	0.0122	0.0122	-	-	N
OTOF	BP1	0.00674	0.0132	0.103	-	-	-	N
	BP2	-	-	-	-	-	-	Y
CDH23	BP1	0.0221	-	0.0227	-	0.00738	-	Y
	BP2	-	-	-	-	-	-	Y
TMC1	BP1	0.000147	0.00925	0.00494	0.00629	0.0469	0.0673	Y
	BP2	0.0299	-	0.0312	-	0.0141	-	Y
KCNQ4	BP1	-	-	-	-	-	-	N
	BP2	-	-	-	-	-	-	N
PCDH15	BP1	0.103	0.140	0.100	0.108	-	-	Y
	BP2	-	-	-	-	-	-	N

Table S6. The number of genes with R > 1 between the echolocating bats and non-echolocating bovids under the US and GS1-4.

Branch pair	US	GS1	GS2	GS3	GS4
**BP1**	328	273	268	218	297
**BP2**	255	205	226	160	224

Table S8. GO enrichment result of genes with R > 1 (*P* value < 0.05) in all combinations of GS1-4 and BPs for echolocating mammals.

Gene	Amino acid change	Functional relations
**GS1, BP1**		
GUCY1B1	(200_NNTT)*	Encodes the beta subunit of the soluble guanylate cyclase (sGC), which produces cGMP in central adaptive auditory responses (Calis, et al. 2023).
ADGRF5	(212_NNSS)	-
NECTIN1	(131_TTAS)	Nectin-1 is crucial for maintaining the checkerboard-like cellular pattern in the cochlear auditory epithelium (Katsunuma, et al. 2022).
TRPV4	(563_VVAA)	Encodes a mechanosensitive non-selective cation channel that is essential for maintaining normal hearing thresholds and protecting the cochlea from acoustic injury (Tabuchi, et al. 2005).
PLXNA1	(421_QQRR)	The *PLXNA1* variant can cause variable sensorineural hearing loss (SNHL) (Dworschak, et al. 2021).
PTCH2	(763_RRQQ,822_MMVV)	-
PTPRB	(1011_IIVV)	A patient with deletion of a genomic region including PTPRB suffers from hearing loss (Alesi, et al. 2017).
P2RX4	(158_TTAS)	Encodes a purinergic receptor subunit that mediates cation absorption in utricular cells together with P2RX2, protecting hair cells from damage during intense auditory stimulation (Jeong, et al. 2020).
GABRR1	(14_IIVV)	-
NTRK2	(37_STFI)	Encodes the high-affinity receptor for brain-derived nerve growth factor (BDNF), which modulates synaptic transmission during the activated status of the auditory system (Gross, et al. 2023).
ADGRF1	(269_NNSS,278_AATT)	-
KIT	(840_AATT)	Encodes a receptor tyrosine kinase, mutations of KIT ligand (KITLG) can cause asymmetric and unilateral hearing loss (Zazo Seco, et al. 2015).
CHRNB2	(292_VVIL)	Encodes a subunit of neuronal acetylcholine receptor with greater than five-fold differential expression in hair cell-enriched compared to the supporting cells (Cristobal, et al. 2005).
ANTXR1	(319_VVLM)	Encodes Anthrax toxin receptor 1, and mutations in *ANTXR1* cause GAPO syndrome, which includes features such as growth retardation, alopecia, pseudoanodontia, and hearing loss (Hamdi, et al. 2024).
GPR160	(193_IIVV)	-
OR51E1	(45_RRWW)	-
LRRC19	(282_GGRK)	-
GRIN2B	(1119_TTSS)	-
TRPM3	(1315_TTAA)	Encodes a transient receptor potential channel that regulates inner ear fluid homeostasis by sensing osmolality changes, thereby maintaining the function of cochlear and vestibular hair cells and protecting against hearing disorders (Takumida, et al. 2009).
PIEZO2	(617_VVAG)	Encodes a mechanosensitive ion channel essential for ultrasonic hearing via cochlear outer hair cells in mice (Li, et al. 2021).
KCNAB3	(92_AATT)	Regulatory subunit of the voltage-gated potassium (Kv) channels in the cochlear nucleus (Friedland, et al. 2007).
LRRC8E	(647_HHNQ)	Encodes a subunit of the volume-regulated anion channel (VRAC) that is essential for maintaining ion homeostasis in the cochlea, and its disruption leads to cochlear degeneration and congenital deafness (Knecht, et al. 2024).

CLCN1	(47_KKTT,109_TTSS)	-
SLC30A3	(185_PPHH)	Encodes ZnT3, a vesicular zinc transporter for loading zinc into vesicles, and is expressed in cochlear hair cells and the spiral limbus (Bizup, et al. 2024).
SLC27A1	(98_AAVV)	Candidate deafness-associated gene in humans (Uetsuka, et al. 2015).
SLC5A6	(552_LLVV)	-
CNNM4	(337_SSNN)	The encoded protein may play a role in metal ion transport and mutations on *CNNM4* cause recessive cone-rod dystrophy (Polok, et al. 2009).
SLC22A4	(15_PPLL)	Encodes an organic cation transporter expressed in the cochlear strial endothelium, essential for maintaining ion homeostasis, and mutations in *SLC22A4* cause recessive non-syndromic hearing loss DFNB60 (Ben Said, et al. 2016).
**GS1, BP2**		
PIK3CG	(19_TTAS)	*PIK3CG* was up-regulated when knock-down *GATA3*, which plays a pivotal role in hearing (Luo, et al. 2013). Subunit of PI3K, which is involved in age-related hearing loss (Garcia-Mato, et al. 2021).
GPR17	(1_TTMM)	-
NET1	(186_HHQQ)	Overexpression of *NET1* promoted cochlear organoid formation and hair cell differentiation (Zhang, et al. 2023).
PRAG1	(1096_VVAA)	-
ATP8B1	(815_IIVV)	Encodes the phosphatidylserine flippase ATP8B1, specifically localized in the stereocilia of cochlear hair cells and essential for their integrity and mechanosensory function, with mutations causing progressive hearing loss through hair cell degeneration (Stapelbroek, et al. 2009).
ABCC6	(446_GGEE,1084_VVLL)	Mutations on *ABCC6* are associated with retinal alteration (Tachikawa, et al. 2008; Martin, et al. 2020).
GJB6	(99_HHQQ)	Encode gap junction protein connexin30, which is expressed in mesenchymal structures of the inner ear (Teubner, et al. 2003). Deficiency of connexin30 caused severe hearing impairment in mice (Teubner, et al. 2003).
AOC2	(259_IIVV)	*AOC2* is presented on the cell surfaces of many tissues, with notably high expression in retina (Horn and Wittung-Stafshede 2021).
ZFHX2	(1011_TTSA,1015_HHQQ)	-
SCNN1G	(491_VVII)	Encodes a subunit of ENaC which essential for maintaining a fluid-free middle ear cavity (Choi, et al. 2006).
SCN11A	(738_IIVV,905_GGRR,990_PPLL,1145_EEKK)	Encodes voltage-gated sodium channels, essential for the development of neurons, deletion of which cause sensorineural hearing loss(Zu, et al. 2021).
SLC17A8	(349_VVLL)	Encodes the vesicular glutamate transporter 3 (VGLUT3) specifically expressed in inner hair cells, essential for neurotransmitter transport and synaptic transmission, with mutations causing autosomal dominant non-syndromic hearing loss DFNA25 (Ryu, et al. 2016).
ABCA4	(979_QNHH,1052_KKEQ)	Mutations on *ABCA4* caused retinal disease (Molday 2007; Cremers, et al. 2020).
PJVK	(99_IIVV)	Required for the proper function of auditory pathway neurons and protects and is essential for the development of auditory hair cells (Defourny, et al. 2019).
GPR179	(750_RRQQ,822_GGRR,1336_LLSS)	Encodes an orphan G protein receptor essential for depolarizing bipolar cell signal transduction in the retina, with mutations

		causing autosomal-recessive congenital stationary night blindness (Peachey, et al. 2012).
STXBP3	(148_IIVV)	Expressed in expressed in epithelial, stromal cells and plays crucial role in sensorineural hearing loss (Ouahed, et al. 2021).
DNASE1L3	(242_RKSG)	-
DDX11	(590_TTMM)	Consistently associated with sensorineural or mixed hearing loss in previous studies (Costales, et al. 2020).
UTP15	(10_TTAA)	Expressed in inner hair cells differentially and under positive selection (Pisciottano, et al. 2019).
ENAM	(24_VVII,179_NNSS)	-
MMP20	(123_VVII)	-
VPS13B	(398_VVII,465_QQHH,1552_TTMM)	-
CDHR1	(37_TTAS,75_VVII)	Mutations on *CDHR1* associated with retinal disease (Duncan, et al. 2012).
MTM1	(135_TTMM)	-
CUL7	(559_QQRR)	Mutations on *CUL7* cause 3M syndrome an autosomal recessive disorder with phenotype of hearing loss (Akalın, et al. 2023).
FAT1	(154_VVII)	*FAT* is a member of atypical cadherins (Vanniya. S, et al. 2018). It involves a pathway called DCHS1-FAT4 signalling pathway which is essential for the development of ear and cochlea (Alaqeel, et al. 2019).
PTPRZ1	(667_HYND)	-
RNF6	(468_RQWW)	-
HECW2	(73_NNSS)	-
TWF2	(173_MMTT)	Encodes a protein that regulates the length of stereocilia during early hair bundle development and is particularly enriched in shorter transducing stereocilia (Carlton, et al. 2021).
NFKBIB	(212_RRWW)	Encodes the inhibitor of the NF-κB signaling pathway, which is up-regulated with age and hearing loss (Tadros, et al. 2008).
SLC20A2	(514_IIVV)	-
MAP7	(565_VVAA)	Encodes a microtubule-associated protein that is expressed in multiple cochlear cell types, particularly in outer hair cells and outer pillar cells, with its expression increasing over time (Lewis, et al. 2022).
HPGD	(25_VVLM)	-
LLGL2	(565_SSNN,763_VVII)	Involved in the signaling pathway and increased Ca^{2+} influx (Leng, et al. 2021).
MYH7B	(401_TTAA)	Encodes an evolutionarily conserved myosin heavy chain expressed in nonmuscle tissues, with mutations linked to hereditary hearing loss (Lee, et al. 2023).
GAS2L1	(162_RRQQ)	-
GAS2L2	(465_TTAP)	Associated with childhood ear infection (Bizaki-Vallaskangas, et al. 2024).
POLD1	(186_TTAA)	Encodes DNA polymerase delta 1, and mutations in *POLD1* can cause deafness .(Zuo, et al. 2022)
TOP3B	(704_IIVV)	-
MCM3	(615_RRQQ)	Highly expressed in Lgr5 cochlear hair cell progenitors, indicating a crucial role in their proliferation and maintenance (Zhang, et al. 2018).
ASCC3	(988_IIVV)	-
POLN	(108_MMTT,333_NNSS)	-
LONP1	(779_VVII)	-
SMC5	(746_NNSS)	-

THRAP3	(274_TTAA)	Patient with mutation in *THRAP3* suffer moderate hearing loss (Clabout, et al. 2023).
HDAC7	(296_VVII)	-
MEF2D	(349_QQPP)	Expressed in the developing inner ear (Sanchez-Calderon, et al. 2010).
ANKRD42	(67_VVAA)	-
CACNB1	(204_QQHH)	-
TRPM6	(783_IITT)	Encodes a transient receptor potential channel highly expressed in the stria vascularis, spiral ligament and prominence, outer and inner hair cells, cochlear supporting cells (Takumida, et al. 2009).
**GS2, BP2**		
SBNO2	(52_TTAA,515_HHRR)	Up-regulated gene in rat cochleae after noise exposure (Yang, et al. 2016).
ITFG2	(374_RRWQ)	-
**GS1, BP1 | GS1, BP2**		
PKD1	(209_VVMM,797_VVTT,1512_VVII,2295_AATT), (545_VVMM,729_AATT,1749_AAVV,2607_AAVV)	Encodes polycystin-1, a large transmembrane protein essential for maintaining the structure and integrity of stereocilia in cochlear hair cells (Steigelman, et al. 2011).
ABCC3	(546_QQKK,924_IIVV,748_HMQQ), (626_IIVV,698_AAVV)	-
**GS1, BP1 | GS3, BP1**		
CDK13	(371_TTII)	Encodes a member of the cyclin-dependent kinase family, which reported associated with sensorineural hearing impairment (Hamilton and Suri 2019).
MOK	(209_EEGA,285_RRSS)	-
CDKL1	(319_CCYY)	-
**GS1, BP2 | GS2, BP2**		
LIPA	(211_IIVV)	-
AXL	(251_GGRR)	-
SCNN1B	(321_MMVV)	Encodes a subunit of ENaC which essential for maintaining a fluid-free middle ear cavity (Choi, et al. 2006).
ADAM11	(79_VVMM)	Encodes a disintegrin and metalloproteinase family protein that is exclusively expressed in amphora-shaped type I hair cells, playing a crucial role in the structural integrity and function of these auditory hair cells (Wang, Ling, et al. 2024).
FYB1	(254_VVMM)	-
LAMA5	(534_TTAA,1020_IIVV,1849_AATT,2112_NNSS,2155_QQRR,2475_RRLL)	Encodes one of the vertebrate laminin alpha chains, whose expression profile changed dynamically in the cochlear epithelial structures (Rodgers, et al. 2001).
LAMC1	(98_IIVV)	Associated with retinal development (Edwards, et al. 2010).
FLNA	(723_IIVV)	Encodes filamin A, and is associated with sensorineural hearing loss (Santos-Cortez, et al. 2021).
CHD7	(1854_TTAA)	Encodes a chromatin remodeler expressed in the endolymphatic sac and duct of the inner ear, with mutations contributing to nonsyndromic hearing loss (Roux, et al. 2023).

SLC39A3	(132_AATT)	-
**GS1, BP2 | GS4, BP2**		
WISP2	(179_TTMM)	Encodes a member of the Wnt signaling pathway that is significantly upregulated in cochleae three days post-implantation (Bas, et al. 2020).
**GS2, BP2 | GS4, BP2**		
CD74	(81_PPSS)	-
CBFA2T3	(393_GGSS,513_AAST), (276_MMVL,393_GGSS,513_AAST)	-
CLEC1B	(40_MMIL)	-
**GS1, BP1 | GS1, BP2 | GS2, BP2**		
ERBB2	(275_SSRR), (143_EEKK)	-
**GS1, BP2 | GS2, BP2 | GS3, BP2**		
KITLG	(178_SSNN)	Mutations of *KITLG* lead to asymmetric and unilateral hearing loss (Zazo Seco, et al. 2015).
GATA1	(336_VVMM)	A paralog of *GATA2* and *GATA3*, which play pivotal roles in hearing (Holley, et al. 2007; Luo, et al. 2013).
EPB42	(278_NNSS)	-
ZBTB7A	(276_AATT)	-
PRKDC	(334_CCGS,691_RQPP,2424_RGEE)	-
MYH9	(1135_AATT)	Encodes a member of myosin family and defect of *MYH9* have been associated with sensorineural hearing loss (Canzi, et al. 2016). Localized to the stereocilia on the inner and outer hair cells in the inner ear in mice (Friedman, et al. 2020).
ITGB6	(3_VVMM)	Encode a subunit beta 6 of integrin, expressed in the mouse developing inner ear and deficient of which cause Stereocilia defects of inner ear (Littlewood Evans and Müller 2000).
MYC	(195_EEAA)	Encodes a transcription factor involved in regulating cell proliferation and differentiation during inner ear development (Domínguez-Frutos, et al. 2011).

* Content within each bracket indicate a convergence event. The number before the underline indicates the site position in the protein sequence alignment. The four amino acids following the underline denote the two amino acid states at the beginning of the two focal branches, and the two amino acid states at the end of the two focal branches.

Table S9. Genes with R > 1 (*P* value < 0.05) in US and GS1-4, and under positive selection in echolocating mammals.

Gene	Grouping scheme	Branch group	Amino acid change	Functional relations
CCDC92	US | GS4	1	(3_TTAA, 108_KKRR)*	*Ccdc92* knockout mice have abnormal auditory brainstem response that might be compatible with a dysfunction of sensory cilia in the cochlea (Duek, et al. 2018).
CDH19	US|GS2|GS4	1	(360_IITT, 371_NNDD)	*CDH19* expressed in cochlea and significantly down-regulated in a middle ear disease (Klenke, et al. 2012; Lin, et al. 2012).
TEX14	US | GS4	1	(77_RRQQ, 452_RRQQ, 756_AATT, 763_DDAA, 912_RRQQ)	*TEX14* has been reported to show 14.5 times higher expression in the cochlea of mice with impending hearing loss than that of wildtype mice, potentially mediating cellular protrusion formation and cytoskeletal organization (Dufek, et al. 2020).
	GS1	1	(77_RRQQ, 292_VVIL, 452_RRQQ, 756_AATT, 763_DDAA, 912_RRQQ)	
	GS2	1	(77_RRQQ, 292_VVIL, 452_RRQQ, 756_AATT, 763_DDAA, 912_RRQQ, 914_TTIP)	
	GS3	1	(77_RRQQ, 452_RRQQ, 763_DDAA, 912_RRQQ)	
CPA3	US	1	(242_RRGG,294_DDEE, 364_PPSS)	*CPA3* is significantly downregulated in hippocampus after acquired hearing loss (Zhou, et al. 2023).
HPGD	US|GS4	1	(66_RRKK)	*HPGD* encodes 15-hydroxyprostaglandin dehydrogenase and is responsible for metabolism of prostaglandins, which in turn affects cochlea function (Nakagawa 2011; Sun, et al. 2025).
WNK3	GS3	2	(26_PPRH)	*WNK3* can dynamically regulate balance between Na-K-2Cl cotransporters (NKCCs) and K-Cl cotransporters (KCCs) activities (Kahle, et al. 2010). Studies have found that inactivation of NKCCs were associated to deafness (Delpire, et al. 1999), and KCC4 expression can be located in the inner ear and loss for KCC3/KCC4 caused deafness (Boettger, et al. 2002; Boettger, et al. 2003).
JPH3	GS4	2	(403_TTAI)	Increased expression during the maturation of mouse primary auditory cortex (Hackett, et al. 2015)
VCAM1	US	2	(593_LLII)	Encodes a cell surface adhesion molecule involved in endothelial function, with elevated levels in sudden sensorineural hearing loss

				patients (Domínguez-Frutos, et al. 2011).
CHAT	US|GS1|GS2|GS3|GS4	2	(114_PPQQ)	Encodes an enzyme responsible for the synthesis of the neurotransmitter acetylcholine, which is significantly decreased in the auditory cortex of *ADNP* mutant mice (Hacohen-Kleiman, et al. 2019).
ELK3	US|GS1|GS2|GS3|GS4	2	(163_QQPP)	-
CYSTM1	US|GS1|GS2|GS4	2	(79_TTPP)	*CYSTM1* is uniquely expressed in the mature basal cell type of inner ear stria vascularis, which is essential to hearing (Thulasiram, et al. 2025).
PRR14L	US|GS1|GS2|GS3|GS4	2	(1076_SSNN)	*PRR14L* is in a deletion that can cause human hearing loss (Trizuljak, et al. 2023).

* Content within each bracket indicates a convergence event. The number before the underline indicates the site position in the protein sequence alignment. The four amino acids following the underline denote the two amino acid states at the beginning of the two focal branches, and the two amino acid states at the end of the two focal branches.

Table S10. The number of sites showing convergence between echolocating bats and non-echolocating bovids, within genes under positive selection in echolocating mammals.

Gene name	Branch pair	Number of convergent sites				
		US	GS1	GS2	GS3	GS4
CCDC92	BP1	0	0	0	0	0
CDH19	BP1	0	0	0	0	0
TEX14	BP1	3	3	3	2	3
CPA3	BP1	0	0	0	0	0
HPGD	BP1	1	0	0	0	1
WNK3	BP2	-	0	-	0	-
JPH3	BP2	0	0	0	0	0
VCAM1	BP2	0	0	0	0	0
CHAT	BP2	0	0	0	0	0
ELK3	BP2	0	0	0	0	0
CYSTM1	BP2	0	0	0	0	0
PRR14L	BP2	0	0	0	0	0

* A dash ('-') indicated that the R-value for the gene was not significant under the respective grouping scheme.

Table S11. Genes with exclusive property convergence and under positive selection in marine mammals.

Gene	*P* value	FDR	Amino acid change	Functional relations
RIBC1	0.016	0.53	(92_VVVVMMLI)*	Though the function of *RIBC1* remains unknown, its important paralog *RIBC2* plays a significant role in the functioning of motile cilia. Respiration system of marine mammals, which contains motile cilia, is quite different from other terrestrial mammals for exchanging of gas efficiently (Kooyman 1973). Enrich in respiratory ciliated cells according to Human Protein Atlas (proteinatlas.org) (Uhlen, et al. 2010).
SWAP70	0.028	0.68	(291_IIMMVVAV)	May influence muscle morphology by regulating the actin cytoskeleton. Related to coronary artery disease, lung immune cell related, and has lung eQTL (Bahaie, et al. 2012; Nikpay, et al. 2015; Qian, et al. 2023).

* Content within each bracket indicate a convergence event. The number before the underline indicates the site position in the protein sequence alignment. The eight amino acids following the underline denote the four amino acid states at the ancestor nodes of the four marine mammals (the ancestral nodes of the two cetaceans are the same), and the four amino acid states of the four marine mammals.

Table S12. Genes with CAAP enriched in "collagen-containing extracellular matrix" for different GSs in marine mammals.

Gene	Amino acid change	Functional relations
**GS1**		
ITIH1	(703_VVVVIIMM)*	Expressed in response to lung injury and promote angiogenesis during cystic fibrosis (Garantziotis, et al. 2008).
**GS2**		
LTBP1	(903_RRRRQQHQ)	Component of extracellular matrix in mouse lung, airway and aorta (Zilberberg, et al. 2012). Influence the formation of elastic fiber and thus may involve in the reconstruction of the respiration system of marine mammals (Jiang, et al. 2016).
COL16A1	(154_VVVASSMT)	Associated with cartilage development in chicken (Takács, et al. 2023).
TINAG	(95_RRRRHHQH)	Component of the core matrisome in lung ECM (Zhou, et al. 2018).
S100A8	(8_IIIIMMMM)	Form heterodimer with *S100A9* and involved in bone and cartilage development in mice (Zreiqat, et al. 2007).
**GS1 | GS2**		
MMP2	(320_VVVVIIII)	Knockout of *MMP2* cause mice to show asthma with allergen expose (Corry, et al. 2002).
**GS1 | GS3**		
SPARC	(228_NNNNKKHH)	Associated with lung cancer and pulmonary fibrosis (Wong and Sukkar 2017). Encode osteonectin, which is a bone-specific protein, thus may play role in the bone system development (Termine, et al. 1981)
LTBP2	(1137_MMMMTTTT)	Associated with lung elastinogenesis and fibrosis (Enomoto, et al. 2018).
**GS1|GS2|GS3**		
LAMA1	(205_QQQQRRRR)	Demonstrated to significantly affect response to lung injury and susceptibility to fibrosis (Lee, et al. 2018).
ERBIN	(438_RRRRQQQQ)	-
SERPINC1	(200_NNNNSSSS)	Regulate blood coagulation; may be associated with low flow rate of viscous blood during diving (Foote, et al. 2015).
ACAN	(357_NNNNSSSS)	Encodes aggrecan, a major component of extracellular matrix essential for the biomechanical properties of cartilage (Shi, et al. 2019).

* Content within each bracket indicate a convergence event. The number before the underline indicates the site position in the protein sequence alignment. The eight amino acids following the underline denote the four amino acid states at the ancestor nodes of the four marine mammals (the ancestral nodes of the two cetaceans are the same), and the four amino acid states of the four marine mammals.

Table S13. The number of genes with CAAP and enriched GO terms in the terrestrial sister species of marine mammals. The '-' means no enriched GO terms or enriched genes were found.

Group scheme	Number of genes with CAAP	Enriched GO terms	Number of enriched genes	FDR
**US**	108	-	-	-
**GS1**	112	aminoacyl-tRNA ligase activity	4	0.041425
		ligase activity, forming carbon-oxygen bonds	4	0.041425
**GS2**	109	-	-	-
**GS3**	64	-	-	-
**GS4**	96	-	-	-

Table S14. Non-repetitive enriched GO terms and involved genes for GS0 in marine mammals.

GO term	FDR	Enriched genes	Functional relations
**Structural molecule activity**	2.40×10^{-5}	*NDC1*	-
		SPTB	-
		LAMA1	Demonstrated to significantly affect response to lung injury and susceptibility to fibrosis (Lee, et al. 2018).
		OLFM4	
		ERBIN	
		LTBP2	Associated with lung elastinogenesis and fibrosis (Enomoto, et al. 2018).
		ACAN	Encodes aggrecan, a major component of extracellular matrix essential for the biomechanical properties of cartilage (Shi, et al. 2019).
		MYL3	-
		KRT19	-
		UMODL1	-
		SYNM	-
**Antiporter activity**	7.60×10^{-4}	*SLC4A4*	-
		CLCN7	-
		SLC26A1	-
		SLC9B1	-

Table S15. Number of convergent genes for mangroves and non-mangrove and the result of KEGG (corresponding to the Fig. 2a in Xu, et al. (2017))

Pathway	Total # of genes in the category	# of convergent genes		*P* value
		mangroves	Non-mangroves	
All	5353	232	117	-
Ubiquitin mediated proteolysis	59	11	0	0.0111 (FDR = 0.737)
Various types of N-glycan biosynthesis	23	5	1	0.0161 (FDR = 0.737)

Table S16. All significantly enriched GO terms under different GS in mangroves.

Grouping scheme	GO term	Ontology class	Number of enriched genes	FDR
GS4	transferase activity, transferring phosphorus-containing groups	MF	13	0.006
GS4	protein kinase activity	MF	12	0.009
GS4	ATP binding	MF	21	0.018
GS4	protein serine/threonine kinase activity	MF	9	0.044
GS4	protein tyrosine kinase activity	MF	6	0.044
GS4	Protein phosphorylation	BP	12	0.041
GS1	ribonucleoside triphosphate phosphatase activity	MF	7	0.030

Table S17. GO and KEGG pathway enrichment result of the gene sets with increased convergent sites in mangroves under GS1 and GS4.

Gene symbol/MSU	Amino acid change	Functional relations
**protein phosphorylation | transferase activity, transferring phosphorus-containing groups | ATP binding (GO term)**		
WNK1	(113_QEEQQQ,238_ISTIIII)*	Transgene of soybean *WNK1* into Arabidopsis significantly enhanced tolerance to NaCl and osmotic stresses (Wang, et al. 2011). Study have found that *WNK1* was regulated by abiotic stress such as salt, heat and involved in maintaining rhythmic behavior in rice (Kumar, et al. 2011).
SPARK2	(29_IFFIIII,427_SRSRRRR, 428_QSNSSSS,436_GEGEEEE)	Downregulated under salt stress in rice (Wang, et al. 2022).
RLCK69	(91_IATTTTT,202_LLMMMMM, 248_EQQEEEE,285_LSSLLLL)	Members of Receptor-like cytoplasmic kinases (RLCK) gene family which play roles in stress response in plants (Vij, et al. 2008).
RLCK172	(36_RKKRRRR,45_SQNSSSS, 47_RKRKKKK,296_PPSSSSS)	Members of Receptor-like cytoplasmic kinases (RLCK) gene family which play roles in stress response in plants (Vij, et al. 2008).
RLCK212	(3_NEQEEEE,190_YYFFFFF, 193_LHLHHHH)	Members of Receptor-like cytoplasmic kinases (RLCK) gene family which play roles in stress response in plants (Vij, et al. 2008).
CDKE1	(394_VIIMMMM,415_PSSPPPP, 420_IMLMMMM)	Mediate the mitochondrial response of Arabidopsis to flooding stress (Meng, et al. 2020). Associated with stress response (Ng, et al. 2013).
EIP10	(191_VVWQQQQ,199_GAAPPPP, 200_CCLMMMM,204_KHHKKKK, 205_AAESSSS,541_GEEGGGG, 548_TVITTTT)	-
LOC_Os03g14710.1	(381_GVAGGGG,425_QQPPPPP)	-
LOC_Os01g67720.1	(3_SLTLLLL,4_SGSGGGG, 61_VLFFFFF)	ABC1-like, ABC genes play roles in salt response in plants (Saha, et al. 2015).
LOC_Os12g24550.1	(213_VSLSSSS,421_KRKRRRR, 510_RILRRRR)	-
LOC_Os03g50390.1	(354_RKRKKKK,546_MILMMMM, 697_KRRKKKK,963_ATTAAAA, 1106_KLAKKKK)	-
**protein phosphorylation | ATP binding (GO term)**		
SMG2	(70_SATAAAA,131_ASTAAAA, 238_TSRRRRR,285_LLQQQQQ, 562_SSNNNNN,756_TASAAAA)	-
**ATP binding | ribonucleoside triphosphate phosphatase activity (GO term)**		
LOC_Os04g21110.1	GS1: (34_TIITTTT,118_NQHHHHH, 248_VIFVVVV,794_VMIVVVV) GS4: (34_TIITTTT,118_NQHHHHH, 716_NDDNNNN)	-
**transferase activity, transferring phosphorus-containing groups (GO term)**		
LOC_Os07g12530.1	(158_STAAAAA,395_NNDDDDD)	-
LOC_Os06g48770.1	(140_DNNDDDD,255_SAASSSS,	-

	491_FALFFFF)	
**ATP binding**		
LOC_Os09g36270.1	(224_HRQQQQQ,469_LLLQQQQ)	-
FTSH8	(108_TSPPPPP,407_FIFIIII, 501_VIMMMMM,532_RKRKKKK, 554_PQPQQQQ)	Plant filamentation temperature-sensitive H (FtsH) proteins was tightly associated with salt stress in *Medicago sativa* L. (Li, Zhu, et al. 2024).
DGK5	(67_APVPPPP,100_TGSGGGG)	Overexpression of *DGK5* enhanced plant susceptibility to salt stress (Li, Yao, et al. 2024).
LOC_Os02g16660.1	(12_KRRKKKK,57_YNNYYYY, 65_IVFFFFF,177_HYYHHHH)	-
TOP2	(739_SGGSSSS,760_SKSNNNN, 807_QLVQQQQ,1044_RSRAAAA)	Play roles in salt response in in Pea and Transgenic Tobacco Plants (Hettiarachchi, et al. 2005).
LOC_Os09g27420.1	(3_IATTTTT,188_NDNDDDD, 390_HHQQQQQ,534_SAASSSS, 624_TASAAAA)	-
MCCB	(353_ATITTTT,361_KRKRRRR)	-
LOC_Os05g41750.1	(27_KEEKKKK,123_AVGGGGG, 233_LMLMMMM)	-
**ribonucleoside triphosphate phosphatase activity (GO term)**		
LOC_Os04g52690.3	(243_ATTAAAA,328_VLIVVVV, 377_VILVVVV)	-
STAR1	(197_KENKKKK,216_VIVIIII)	Bind to ASR5 protein which play roles in stress response (Arenhart, et al. 2014; Arenhart, et al. 2016).
ABCF6	(159_LLVVVVV,557_NKEKKKK)	Member of ABC family, ABC genes play roles in salt response in plants (Saha, et al. 2015).
LOC_Os06g01980.1	(119_LPLPPPP,239_NSDSSSS, 244_PLFPPPP)	-
ABCI14	(69_SNGNNNN,92_TSTSSSS, 122_GIAVVVV)	Member of ABC family, ABC genes play roles in salt response in plants (Saha, et al. 2015).
LOC_Os03g15810.1	(47_TAPTTTT,147_IVVIIII, 222_RQQRRRR,223_IITTTTT)	-
**phosphatidylinositol signaling system (KEGG pathway)**		
DGK5	(67_APVPPPP,100_TGSGGGG)	Core enzyme in plant polyphosphoinositide metabolism responsing to osmotic stress (Munnik and Vermeer 2010).
PLC2	(230_RDHKKKK,339_NKNKKKK)	Core enzyme in plant polyphosphoinositide metabolism responsing to osmotic stress (Munnik and Vermeer 2010).
LOC_Os03g48300.1	(65_FYYFFFF,407_DGDGGGG, 617_EKDKKKK,835_KRRKKKK)	Downstream gene involved in InsP_6 synthesis (Wang, Cui, et al. 2024).

* Content within each bracket indicate a convergence event. The number before the underline indicates the site position in the protein sequence alignment. The seven amino acids following the underline denote: (1-3) the amino acid states of the three mangrove species; (4-6) the amino acid states of the three non-mangrove species; (7) the amino acid states of the outgroup.

References

Akalın A, Şimşek-Kiper PÖ, Taşkıran E, Utine GE, Boduroğlu K. 2023. Typical Face, Developmental Delay, and Hearing Loss in a Patient with 3M Syndrome: The Co-Occurrence of Two Rare Conditions. Molecular Syndromology 13:537-542.

Alaqeel B, Babiker A, Al Mutairi F, Al Dubayee M. 2019. Coexistence of genetic conditions: exploring a possible relationship. Sudan J Paediatr 19:60-66.

Alesi V, Loddo S, Grispo M, Riccio S, Montella AC, Dallapiccola B, Ulgheri L, Novelli AJEJoMG. 2017. Reassessment of the 12q15 deletion syndrome critical region. 60:220-223.

Arenhart RA, Bai Y, Valter de Oliveira LF, Bucker Neto L, Schunemann M, Maraschin FdS, Mariath J, Silverio A, Sachetto-Martins G, Margis R, et al. 2014. New Insights into Aluminum Tolerance in Rice: The ASR5 Protein Binds the STAR1 Promoter and Other Aluminum-Responsive Genes. Molecular Plant 7:709-721.

Arenhart RA, Schunemann M, Bucker Neto L, Margis R, Wang Z-Y, Margis-Pinheiro M. 2016. Rice ASR1 and ASR5 are complementary transcription factors regulating aluminium responsive genes. Plant, Cell & Environment 39:645-651.

Bahaie NS, Hosseinkhani MR, Ge XN, Kang BN, Ha SG, Blumenthal MS, Jessberger R, Rao SP, Sriramarao P. 2012. Regulation of Eosinophil Trafficking by SWAP-70 and Its Role in Allergic Airway Inflammation. The Journal of Immunology 188:1479-1490.

Bas E, Anwar MR, Van De Water TR. 2020. TGF β-1 and WNT Signaling Pathways Collaboration Associated with Cochlear Implantation Trauma-Induced Fibrosis. The Anatomical Record 303:608-618.

Ben Said M, Grati Mh, Ishimoto T, Zou B, Chakchouk I, Ma Q, Yao Q, Hammami B, Yan D, Mittal R, et al. 2016. A mutation in SLC22A4 encoding an organic cation transporter expressed in the cochlea strial endothelium causes human recessive non-syndromic hearing loss DFNB60. Human Genetics 135:513-524.

Bizaki-Vallaskangas A, Rämö J, Sliz E, Kivekäs I, Willberg T, Saarentaus E, Toppila-Salmi S, Dietz A, Haapaniemi T, Hytönen VP, et al. 2024. Genome-wide association study indicates novel associations of annexin A13 to secretory and GAS2L2 with mucous otitis media. Scientific Reports 14:18344.

Bizup B, Brutsaert S, Cunningham CL, Thathiah A, Tzounopoulos T. 2024. Cochlear zinc signaling dysregulation is associated with noise-induced hearing loss, and zinc chelation enhances cochlear recovery. 121:e2310561121.

Boettger T, Hübner CA, Maier H, Rust MB, Beck FX, Jentsch TJ. 2002. Deafness and renal tubular acidosis in mice lacking the K-Cl co-transporter Kcc4. Nature 416:874-878.

Boettger T, Rust MB, Maier H, Seidenbecher T, Schweizer M, Keating DJ, Faulhaber J, Ehmke H, Pfeffer C, Scheel O, et al. 2003. Loss of K-Cl co-transporter KCC3 causes deafness, neurodegeneration and reduced seizure threshold. The EMBO Journal 22:5422-5434-5434.

Calis D, Hess M, Marchetta P, Singer W, Modro J, Nelissen E, Prickaerts J, Sandner P, Lukowski R, Ruth PJFimn. 2023. Acute deletion of the central MR/GR steroid receptor correlates with changes in LTP, auditory neural gain, and GC-A cGMP signaling. 16:1017761.

Canzi P, Pecci A, Manfrin M, Rebecchi E, Zaninetti C, Bozzi V, Benazzo M. 2016. Severe to profound deafness may be associated with MYH9-related disease: report of 4 patients. Acta Otorhinolaryngol Ital 36:415-420.

Carlton AJ, Halford J, Underhill A, Jeng J-Y, Avenarius MR, Gilbert ML, Ceriani F, Ebisine K, Brown SDM, Bowl MR, et al. 2021. Loss of Baiap2l2 destabilizes the transducing stereocilia of cochlear hair cells and leads to deafness. 599:1173-1198.

Choi JY, Son EJ, Kim JL, Lee J-H, Park HY, Kim SH, Song MH, Yoon J-H. 2006. ENaC- and CFTR-dependent ion and fluid transport in human middle ear epithelial cells. Hearing Research 211:26-32.

Clabout T, Maes L, Acke F, Wuyts W, Van Schil K, Coucke P, Janssens S, De Leenheer E. 2023. Negative Molecular Diagnostics in Non-Syndromic Hearing Loss: What Next? 14:105.

Corry DB, Rishi K, Kanellis J, Kiss A, Song L-z, Xu J, Feng L, Werb Z, Kheradmand F. 2002. Decreased allergic lung inflammatory cell egression and increased susceptibility to asphyxiation in MMP2-deficiency. Nature Immunology 3:347-353.

Costales M, Diñeiro M, Cifuentes GÁ, Capín R, Otero A, Viejo-Díaz M, Plasencia A, Núñez F, Gómez JR, Llorente JL, et al. 2020. Clinical Utility of Next-generation Sequencing in the Aetiological Diagnosis of

Sensorineural Hearing Loss in a Childhood Hearing Loss Unit. Acta Otorrinolaringologica (English Edition) 71:166-174.
Cremers FPM, Lee W, Collin RWJ, Allikmets R. 2020. Clinical spectrum, genetic complexity and therapeutic approaches for retinal disease caused by ABCA4 mutations. Progress in Retinal and Eye Research 79:100861.
Cristobal R, Wackym PA, Cioffi JA, Erbe CB, Roche JP, Popper PJMbr. 2005. Assessment of differential gene expression in vestibular epithelial cell types using microarray analysis. 133:19-36.
Defourny J, Aghaie A, Perfettini I, Avan P, Delmaghani S, Petit C. 2019. Pejvakin-mediated pexophagy protects auditory hair cells against noise-induced damage. Proceedings of the National Academy of Sciences 116:8010-8017.
Delpire E, Lu J, England R, Dull C, Thorne T. 1999. Deafness and imbalance associated with inactivation of the secretory Na-K-2Cl co-transporter. Nature Genetics 22:192-195.
Domínguez-Frutos E, López-Hernández I, Vendrell V, Neves J, Gallozzi M, Gutsche K, Quintana L, Sharpe J, Knoepfler PS, Eisenman RN, et al. 2011. N-myc Controls Proliferation, Morphogenesis, and Patterning of the Inner Ear. The Journal of Neuroscience 31:7178.
Duek P, Gateau A, Bairoch A, Lane L. 2018. Exploring the Uncharacterized Human Proteome Using neXtProt. J Proteome Res 17:4211-4226.
Dufek B, Meehan DT, Delimont D, Samuelson G, Madison J, Shi X, Boettcher F, Trosky V, Gratton MA, Cosgrove D. 2020. Pericyte abnormalities precede strial capillary basement membrane thickening in Alport mice. Hear Res 390:107935.
Duncan JL, Roorda A, Navani M, Vishweswaraiah S, Syed R, Soudry S, Ratnam K, Gudiseva HV, Lee P, Gaasterland T, et al. 2012. Identification of a Novel Mutation in the CDHR1 Gene in a Family With Recessive Retinal Degeneration. Archives of Ophthalmology 130:1301-1308.
Dworschak GC, Punetha J, Kalanithy JC, Mingardo E, Erdem HB, Akdemir ZC, Karaca E, Mitani T, Marafi D, Fatih JM, et al. 2021. Biallelic and monoallelic variants in PLXNA1 are implicated in a novel neurodevelopmental disorder with variable cerebral and eye anomalies. Genetics in Medicine 23:1715-1725.
Edwards MM, Mammadova-Bach E, Alpy F, Klein A, Hicks WL, Roux M, Simon-Assmann P, Smith RS, Orend G, Wu J, et al. 2010. Mutations in Lama1 Disrupt Retinal Vascular Development and Inner Limiting Membrane Formation. Journal of Biological Chemistry 285:7697-7711.
Enomoto Y, Matsushima S, Shibata K, Aoshima Y, Yagi H, Meguro S, Kawasaki H, Kosugi I, Fujisawa T, Enomoto N, et al. 2018. LTBP2 is secreted from lung myofibroblasts and is a potential biomarker for idiopathic pulmonary fibrosis. Clinical Science 132:1565-1580.
Foote AD, Liu Y, Thomas GWC, Vinař T, Alföldi J, Deng J, Dugan S, van Elk CE, Hunter ME, Joshi V, et al. 2015. Convergent evolution of the genomes of marine mammals. Nature Genetics 47:272-275.
Friedland DR, Eernisse R, Popper P. 2007. Potassium channel gene expression in the rat cochlear nucleus. Hearing Research 228:31-43.
Friedman TB, Belyantseva IA, Frolenkov GI. 2020. Myosins and Hearing. In: Coluccio LM, editor. Myosins: A Superfamily of Molecular Motors. Cham: Springer International Publishing. p. 317-330.
Garantziotis S, Zudaire E, Trempus CS, Hollingsworth JW, Jiang D, Lancaster LH, Richardson E, Zhuo L, Cuttitta F, Brown KK, et al. 2008. Serum Inter–α-Trypsin Inhibitor and Matrix Hyaluronan Promote Angiogenesis in Fibrotic Lung Injury. American Journal of Respiratory and Critical Care Medicine 178:939-947.
Garcia-Mato A, Cervantes B, Murillo-Cuesta S, Rodriguez-de la Rosa L, Varela-Nieto I. 2021. Insulin-like Growth Factor 1 Signaling in Mammalian Hearing. Genes (Basel) 12.
Gross J, Knipper M, Mazurek BJC, Neurobiology M. 2023. Candidate Key Proteins in Tinnitus: A Bioinformatic Study of Synaptic Transmission in Spiral Ganglion Neurons. 43:4189-4207.
Hackett TA, Guo Y, Clause A, Hackett NJ, Garbett K, Zhang P, Polley DB, Mirnics K. 2015. Transcriptional maturation of the mouse auditory forebrain. BMC Genomics 16:606.
Hacohen-Kleiman G, Yizhar-Barnea O, Touloumi O, Lagoudaki R, Avraham KB, Grigoriadis N, Gozes I. 2019. Atypical Auditory Brainstem Response and Protein Expression Aberrations Related to ASD and Hearing

Loss in the Adnp Haploinsufficient Mouse Brain. Neurochemical Research 44:1494-1507.
Hamdi M, Alkheder A, Hammoud KJ, Issa MY, ALGhabra Y, Yousfan AJE, Nose, Journal T. 2024. Novel Otolaryngological and Radiological Manifestations in GAPO Syndrome.01455613241307753.
Hamilton MJ, Suri M. 2019. Chapter Five - CDK13-related disorder. In: Kumar D, editor. Advances in Genetics: Academic Press. p. 163-182.
Hettiarachchi GHCM, Reddy MK, Sopory SK, Chattopadhyay S. 2005. Regulation of TOP2 by Various Abiotic Stresses Including Cold and Salinity in Pea and Transgenic Tobacco Plants. Plant and Cell Physiology 46:1154-1160.
Holley MC, Kneebone A, Milo M. 2007. Information for gene networks in inner ear development: A study centered on the transcription factor gata2. Hear Res 227:32-40.
Horn N, Wittung-Stafshede PJB. 2021. ATP7A-regulated enzyme metalation and trafficking in the menkes disease puzzle. 9:391.
Jeong J, Kim JY, Hong H, Wangemann P, Marcus DC, Jung J, Choi JY, Kim SH. 2020. P2RX2 and P2RX4 receptors mediate cation absorption in transitional cells and supporting cells of the utricular macula. Hearing Research 386:107860.
Jiang Y, Feng S, Xu J, Zhang S, Li S, Sun X, Xu P. 2016. Comparative transcriptome analysis between aquatic and aerial breathing organs of Channa argus to reveal the genetic basis underlying bimodal respiration. Marine Genomics 29:89-96.
Kahle KT, Rinehart J, Lifton RP. 2010. Phosphoregulation of the Na–K–2Cl and K–Cl cotransporters by the WNK kinases. Biochimica et Biophysica Acta (BBA) - Molecular Basis of Disease 1802:1150-1158.
Katsunuma S, Togashi H, Kuno S, Fujita T, Nibu K-IJFiC, Biology D. 2022. Hearing loss in mice with disruption of auditory epithelial patterning in the cochlea. 10:1073830.
Klenke C, Janowski S, Borck D, Widera D, Ebmeyer J, Kalinowski J, Leichtle A, Hofestädt R, Upile T, Kaltschmidt C, et al. 2012. Identification of Novel Cholesteatoma-Related Gene Expression Signatures Using Full-Genome Microarrays. PLOS ONE 7:e52718.
Knecht DA, Zeziulia M, Bhavsar MB, Puchkov D, Maier H, Jentsch TJJJoBC. 2024. LRRC8/VRAC volume-regulated anion channels are crucial for hearing. 300.
Kooyman GL. 1973. Respiratory Adaptations in Marine Mammals. American Zoologist 13:457-468.
Kumar K, Rao KP, Biswas DK, Sinha AK. 2011. Rice WNK1 is regulated by abiotic stress and involved in internal circadian rhythm. Plant Signaling & Behavior 6:316-320.
Lee C-M, Cho SJ, Cho W-K, Park JW, Lee J-H, Choi AM, Rosas IO, Zheng M, Peltz G, Lee CG, et al. 2018. Laminin α1 is a genetic modifier of TGF-β1–stimulated pulmonary fibrosis. JCI Insight 3.
Lee LA, Barrick SK, Buvoli AE, Walklate J, Stump WT, Geeves M, Greenberg MJ, Leinwand LA. 2023. Distinct effects of two hearing loss–associated mutations in the sarcomeric myosin MYH7b. Journal of Biological Chemistry 299.
Leng S, Xie F, Liu J, Shen J, Quan G, Wen T. 2021. LLGL2 Increases Ca2+ Influx and Exerts Oncogenic Activities via PI3K/AKT Signaling Pathway in Hepatocellular Carcinoma. 11.
Lewis MA, Ingham NJ, Chen J, Pearson S, Di Domenico F, Rekhi S, Allen R, Drake M, Willaert A, Rook V, et al. 2022. Identification and characterisation of spontaneous mutations causing deafness from a targeted knockout programme. BMC Biology 20:67.
Li J, Liu S, Song C, Hu Q, Zhao Z, Deng T, Wang Y, Zhu T, Zou L, Wang S, et al. 2021. PIEZO2 mediates ultrasonic hearing via cochlear outer hair cells in mice. 118:e2101207118.
Li J, Yao S, Kim S-C, Wang X. 2024. Lipid phosphorylation by a diacylglycerol kinase suppresses ABA biosynthesis to regulate plant stress responses. Molecular Plant 17:342-358.
Li M, Zhu X, Yu Q, Yu A, Chen L, Kang J, Wang X, Yang T, Yang Q, Long R. 2024. FtsH proteases confer protection against salt and oxidative stress in Medicago sativa L. Plant Science 338:111915.
Lin J, Yan X, Wang C, Guo Z, Rolfs A, Luo J. 2012. Anatomical expression patterns of delta-protocadherins in developing chicken cochlea. Journal of Anatomy 221:598-608.
Littlewood Evans A, Müller U. 2000. Stereocilia defects in the sensory hair cells of the inner ear in mice deficient in integrin α8β1. Nature Genetics 24:424-428.
Luo X-j, Deng M, Xie X, Huang L, Wang H, Jiang L, Liang G, Hu F, Tieu R, Chen R, et al. 2013. GATA3

controls the specification of prosensory domain and neuronal survival in the mouse cochlea. Human Molecular Genetics 22:3609-3623.
Martin G, Isabel W, Johannes B, Philipp LM, Isabel F, Doris H, Frank GH, Robert PF, Peter Charbel I. 2020. Retinal findings in carriers of monoallelic ABCC6 mutations. British Journal of Ophthalmology 104:1089.
Meng X, Li L, Narsai R, De Clercq I, Whelan J, Berkowitz O. 2020. Mitochondrial signalling is critical for acclimation and adaptation to flooding in Arabidopsis thaliana. The Plant Journal 103:227-247.
Molday RS. 2007. ATP-binding cassette transporter ABCA4: Molecular properties and role in vision and macular degeneration. Journal of Bioenergetics and Biomembranes 39:507-517.
Munnik T, Vermeer JE. 2010. Osmotic stress-induced phosphoinositide and inositol phosphate signalling in plants. Plant, Cell & Environment 33:655-669.
Nakagawa T. 2011. Roles of prostaglandin E2 in the cochlea. Hear Res 276:27-33.
Ng S, Giraud E, Duncan O, Law SR, Wang Y, Xu L, Narsai R, Carrie C, Walker H, Day DA, et al. 2013. Cyclin-dependent Kinase E1 (CDKE1) Provides a Cellular Switch in Plants between Growth and Stress Responses. Journal of Biological Chemistry 288:3449-3459.
Nikpay M, Goel A, Won H-H, Hall LM, Willenborg C, Kanoni S, Saleheen D, Kyriakou T, Nelson CP, Hopewell JC, et al. 2015. A comprehensive 1000 Genomes–based genome-wide association meta-analysis of coronary artery disease. Nature Genetics 47:1121-1130.
Ouahed J, Kelsen JR, Spessott WA, Kooshesh K, Sanmillan ML, Dawany N, Sullivan KE, Hamilton KE, Slowik V, Nejentsev S, et al. 2021. Variants in STXBP3 are Associated with Very Early Onset Inflammatory Bowel Disease, Bilateral Sensorineural Hearing Loss and Immune Dysregulation. Journal of Crohn's and Colitis 15:1908-1919.
Peachey Neal S, Ray Thomas A, Florijn R, Rowe Lucy B, Sjoerdsma T, Contreras-Alcantara S, Baba K, Tosini G, Pozdeyev N, Iuvone PM, et al. 2012. GPR179 Is Required for Depolarizing Bipolar Cell Function and Is Mutated in Autosomal-Recessive Complete Congenital Stationary Night Blindness. The American Journal of Human Genetics 90:331-339.
Pisciottano F, Cinalli AR, Stopiello JM, Castagna VC, Elgoyhen AB, Rubinstein M, Gómez-Casati ME, Franchini LF. 2019. Inner Ear Genes Underwent Positive Selection and Adaptation in the Mammalian Lineage. Molecular Biology and Evolution 36:1653-1670.
Polok B, Escher P, Ambresin A, Chouery E, Bolay S, Meunier I, Nan F, Hamel C, Munier FL, Thilo B, et al. 2009. Mutations in CNNM4 cause recessive cone-rod dystrophy with amelogenesis imperfecta. Am J Hum Genet 84:259-265.
Qian Q, Hu F, Yu W, Leng D, Li Y, Shi H, Deng D, Ding K, Liang C, Liu J. 2023. SWAP70 Overexpression Protects Against Pathological Cardiac Hypertrophy in a TAK1-Dependent Manner. Journal of the American Heart Association 12:e028628.
Rodgers KD, Barritt L, Miner JH, Cosgrove D. 2001. The laminins in the murine inner ear: developmental transitions and expression in cochlear basement membranes. Hearing Research 158:39-50.
Roux I, Fenollar-Ferrer C, Lee HJ, Chattaraj P, Lopez IA, Han K, Honda K, Brewer CC, Butman JA, Morell RJ, et al. 2023. CHD7 variants associated with hearing loss and enlargement of the vestibular aqueduct. Human Genetics 142:1499-1517.
Ryu N, Sagong B, Park H-J, Kim M-A, Lee K-Y, Choi JY, Kim U-K. 2016. Screening of the SLC17A8 gene as a causative factor for autosomal dominant non-syndromic hearing loss in Koreans. BMC Medical Genetics 17:6.
Saha J, Sengupta A, Gupta K, Gupta B. 2015. Molecular phylogenetic study and expression analysis of ATP-binding cassette transporter gene family in Oryza sativa in response to salt stress. Computational Biology and Chemistry 54:18-32.
Sanchez-Calderon H, Rodriguez-de la Rosa L, Milo M, Pichel JG, Holley M, Varela-Nieto I. 2010. RNA Microarray Analysis in Prenatal Mouse Cochlea Reveals Novel IGF-I Target Genes: Implication of MEF2 and FOXM1 Transcription Factors. PLOS ONE 5:e8699.
Santos-Cortez RLP, Yarza TKL, Bootpetch TC, Tantoco MLC, Mohlke KL, Cruz TLG, Chiong Perez ME, Chan AL, Lee NR, Tobias-Grasso CAM, et al. 2021. Identification of Novel Candidate Genes and Variants for Hearing Loss and Temporal Bone Anomalies. Genes. doi: 10.3390/genes12040566

Shi Y, Hu X, Cheng J, Zhang X, Zhao F, Shi W, Ren B, Yu H, Yang P, Li Z, et al. 2019. A small molecule promotes cartilage extracellular matrix generation and inhibits osteoarthritis development. Nature Communications 10:1914.
Stapelbroek JM, Peters TA, van Beurden DHA, Curfs JHAJ, Joosten A, Beynon AJ, van Leeuwen BM, van der Velden LM, Bull L, Oude Elferink RP, et al. 2009. ATP8B1 is essential for maintaining normal hearing. 106:9709-9714.
Steigelman KA, Lelli A, Wu X, Gao J, Lin S, Piontek K, Wodarczyk C, Boletta A, Kim H, Qian F, et al. 2011. Polycystin-1 Is Required for Stereocilia Structure But Not for Mechanotransduction in Inner Ear Hair Cells. The Journal of Neuroscience 31:12241.
Sun M, Liu Y, Wang X, Wang L. 2025. HPGD: An Intermediate Player in Microglial Polarization and Multiple Sclerosis Regulated by Nr4a1. Mol Neurobiol 62:271-287.
Tabuchi K, Suzuki M, Mizuno A, Hara A. 2005. Hearing impairment in TRPV4 knockout mice. Neuroscience Letters 382:304-308.
Tachikawa M, Toki H, Tomi M, Hosoya K-i. 2008. Gene expression profiles of ATP-binding cassette transporter A and C subfamilies in mouse retinal vascular endothelial cells. Microvascular Research 75:68-72.
Tadros SF, D'Souza M, Zhu X, Frisina RD. 2008. Apoptosis-related genes change their expression with age and hearing loss in the mouse cochlea. Apoptosis 13:1303-1321.
Takács R, Vágó J, Póliska S, Pushparaj Peter N, Ducza L, Kovács P, Jin E-J, Barrett-Jolley R, Zákány R, Matta C. 2023. The temporal transcriptomic signature of cartilage formation. Nucleic Acids Research 51:3590-3617.
Takumida M, Ishibashi T, Hamamoto T, Hirakawa K, Anniko M. 2009. Expression of transient receptor potential channel melastin (TRPM) 1–8 and TRPA1 (ankyrin) in mouse inner ear. Acta Oto-Laryngologica 129:1050-1060.
Termine JD, Kleinman HK, Whitson SW, Conn KM, McGarvey ML, Martin GR. 1981. Osteonectin, a bone-specific protein linking mineral to collagen. Cell 26:99-105.
Teubner B, Michel V, Pesch J, Lautermann J, Cohen-Salmon M, Söhl G, Jahnke K, Winterhager E, Herberhold C, Hardelin J-P, et al. 2003. Connexin30 (Gjb6)-deficiency causes severe hearing impairment and lack of endocochlear potential. Human Molecular Genetics 12:13-21.
Thulasiram MR, Yamamoto R, Olszewski RT, Gu S, Morell RJ, Hoa M, Dabdoub A. 2025. Molecular differences between young and mature stria vascularis from organotypic explants and transcriptomics. iScience 28:111832.
Trizuljak J, Duben J, Blaháková I, Vrzalová Z, Kozubík KS, Štika J, Radová L, Bergerová V, Mejstříková S, Hořínová V, et al. 2023. Extensive, 3.8 Mb-Sized Deletion of 22q12 in a Patient with Bilateral Schwannoma, Intellectual Disability, Sensorineural Hearing Loss, and Epilepsy. Molecular Syndromology 14:439-448.
Uetsuka S, Ogata G, Nagamori S, Isozumi N, Nin F, Yoshida T, Komune S, Kitahara T, Kikkawa Y, Inohara H, et al. 2015. Molecular architecture of the stria vascularis membrane transport system, which is essential for physiological functions of the mammalian cochlea. 42:1984-2002.
Uhlen M, Oksvold P, Fagerberg L, Lundberg E, Jonasson K, Forsberg M, Zwahlen M, Kampf C, Wester K, Hober S, et al. 2010. Towards a knowledge-based Human Protein Atlas. Nat Biotechnol 28:1248-1250.
Vanniya. S P, Srisailapathy CRS, Kunka Mohanram R. 2018. The tip link protein Cadherin-23: From Hearing Loss to Cancer. Pharmacological Research 130:25-35.
Vij S, Giri J, Dansana PK, Kapoor S, Tyagi AK. 2008. The Receptor-Like Cytoplasmic Kinase (OsRLCK) Gene Family in Rice: Organization, Phylogenetic Relationship, and Expression during Development and Stress. Molecular Plant 1:732-750.
Wang J, Li Y, Wang Y, Du F, Zhang Y, Yin M, Zhao X, Xu J, Yang Y, Wang W, et al. 2022. Transcriptome and Metabolome Analyses Reveal Complex Molecular Mechanisms Involved in the Salt Tolerance of Rice Induced by Exogenous Allantoin. Antioxidants. doi: 10.3390/antiox11102045
Wang L, Cui J, Zhang N, Wang X, Su J, Vallés MP, Wu S, Yao W, Chen X, Chen D. 2024. OsIPK1 frameshift mutations disturb phosphorus homeostasis and impair starch synthesis during grain filling in rice. Plant Molecular Biology 114:91.

Wang T, Ling AH, Billings SE, Hosseini DK, Vaisbuch Y, Kim GS, Atkinson PJ, Sayyid ZN, Aaron KA, Wagh D, et al. 2024. Single-cell transcriptomic atlas reveals increased regeneration in diseased human inner ear balance organs. Nature Communications 15:4833.
Wang Y, Suo H, Zhuang C, Ma H, Yan X. 2011. Overexpression of the soybean GmWNK1 altered the sensitivity to salt and osmotic stress in Arabidopsis. Journal of Plant Physiology 168:2260-2267.
Wong SLI, Sukkar MB. 2017. The SPARC protein: an overview of its role in lung cancer and pulmonary fibrosis and its potential role in chronic airways disease. British Journal of Pharmacology 174:3-14.
Xu S, He Z, Guo Z, Zhang Z, Wyckoff GJ, Greenberg A, Wu C-I, Shi S. 2017. Genome-Wide Convergence during Evolution of Mangroves from Woody Plants. Molecular Biology and Evolution 34:1008-1015.
Yang S, Cai Q, Vethanayagam RR, Wang J, Yang W, Hu BH. 2016. Immune defense is the primary function associated with the differentially expressed genes in the cochlea following acoustic trauma. Hearing Research 333:283-294.
Zazo Seco C, Serrão de Castro L, van Nierop Josephine W, Morín M, Jhangiani S, Verver Eva JJ, Schraders M, Maiwald N, Wesdorp M, Venselaar H, et al. 2015. Allelic Mutations of KITLG, Encoding KIT Ligand, Cause Asymmetric and Unilateral Hearing Loss and Waardenburg Syndrome Type 2. The American Journal of Human Genetics 97:647-660.
Zhang L, Fang Y, Tan F, Guo F, Zhang Z, Li N, Sun Q, Qi J, Chai R. 2023. AAV-Net1 facilitates the trans-differentiation of supporting cells into hair cells in the murine cochlea. Cellular and Molecular Life Sciences 80:86.
Zhang Y, Guo L, Lu X, Cheng C, Sun S, Li W, Zhao L, Lai C, Zhang S, Yu C, et al. 2018. Characterization of Lgr6+ Cells as an Enriched Population of Hair Cell Progenitors Compared to Lgr5+ Cells for Hair Cell Generation in the Neonatal Mouse Cochlea. 11.
Zhou X, Jin L, Li Y, Wang Y, Li W, Shen X. 2023. Comprehensive analysis of N6-methyladenosine-related RNA methylation in the mouse hippocampus after acquired hearing loss. BMC Genomics 24:577.
Zhou Y, Horowitz JC, Naba A, Ambalavanan N, Atabai K, Balestrini J, Bitterman PB, Corley RA, Ding B-S, Engler AJ, et al. 2018. Extracellular matrix in lung development, homeostasis and disease. Matrix Biology 73:77-104.
Zilberberg L, Todorovic V, Dabovic B, Horiguchi M, Couroussé T, Sakai LY, Rifkin DB. 2012. Specificity of latent TGF-β binding protein (LTBP) incorporation into matrix: Role of fibrillins and fibronectin. Journal of Cellular Physiology 227:3828-3836.
Zreiqat H, Howlett CR, Gronthos S, Hume D, Geczy CL. 2007. S100A8/S100A9 and their association with cartilage and bone. Journal of Molecular Histology 38:381-391.
Zu M, Guo W-W, Cong T, Ji F, Zhang S-L, Zhang Y, Song X, Sun W, He DZZ, Shi W-G, et al. 2021. SCN11A gene deletion causes sensorineural hearing loss by impairing the ribbon synapses and auditory nerves. BMC Neuroscience 22:18.
Zuo B, Xu H, Pan Z, Mao L, Feng H, Zeng B, Tang W, Lu W. 2022. A likely pathogenic POLD1 variant associated with mandibular hypoplasia, deafness, progeroid features, and lipodystrophy syndrome in a Chinese patient. BMC Medical Genomics 15:220.
